# Cooperation of Two Metal Centers in a CO_2_ Electroreduction Catalyst: Flexible Electron Manipulation and Adaptive Coordination on a Dinuclear Cobalt Complex

**DOI:** 10.1002/advs.202508361

**Published:** 2025-10-14

**Authors:** Yunyi Pan, Masaki Donoshita, Yohei Kametani, Yoshihito Shiota, Shu‐Qi Wu, Osamu Sato, Miho Yamauchi

**Affiliations:** ^1^ Department of Chemistry Graduate School of Science Kyushu University Fukuoka 819‐0395 Japan; ^2^ Institute for Materials Chemistry and Engineering (IMCE) Kyushu University Fukuoka 819‐0395 Japan; ^3^ Department of Applied Chemistry Graduate School of Engineering Kyushu University Fukuoka 819‐0395 Japan; ^4^ International Institute for Carbon‐Neutral Energy Research (WPI‐I^2^CNER) Kyushu University Fukuoka 819‐0395 Japan; ^5^ Research Center for Negative Emissions Technologies (K‐NETs) Kyushu University Fukuoka 819‐0395 Japan; ^6^ Advanced Institute for Materials Research (WPI‐AIMR) Tohoku University Sendai 980‐8577 Japan

**Keywords:** CO_2_ electrolysis, cobalt, DFT calculations, dinuclear metal complex, redox

## Abstract

Metal complexes with flexible redox and coordination properties possibly act as ingenious electrocatalysts to activate stable molecules such as CO_2_. To date, most studies have focused on mononuclear complexes in electrochemical CO_2_ reduction (eCO_2_R). However, the development of electrocatalysts that operate at low overpotentials while maintaining high selectivity remains a critical challenge. Herein, it is demonstrated that the use of a dinuclear metal complex exhibiting cooperation of two metal centers can be an effective strategy for addressing this issue. The focused catalyst is a Co^II^ dinuclear complex (**2**) bearing two Co^II^ ions in close proximity, whose coordination environment is similar to that of a typical mononuclear complex, Co^II^ tetraphenylporphyrin (**1**). Based on experimental and computational studies, it is clarified that **2** exhibits simultaneous two‐electron reduction prior to CO_2_ activation, which allows bypassing the reaction step that hinders the catalytic cycle on **1**. Furthermore, metal‐to‐metal electron transfer and a CO_2_‐derived intermediate bridging over two metal centers are found in the catalytic cycle of **2**, which would contribute to low activation barrier. It is then concluded that the cooperative functions of the two metal centers are the key to the efficient eCO_2_R performance.

## Graphical abstract

It is demonstrated that a dinuclear cobalt complex with two metal centers located in close proximity efficiently catalyzes CO_2_ electroreduction. Based on the experimental and computational studies, the reason for the efficient catalytic performance is attributed to the cooperation of two metal centers, i.e., simultaneous two‐electron accumulation, metal‐to‐metal electron transfer, and a bridging intermediate.

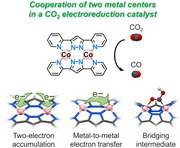

## 1 Introduction

Electrochemical CO_2_ reduction (eCO_2_R), which provides valuable chemicals from CO_2_ and electricity, has attracted growing attention as a solution to the environmental and energy problems.^[^ ^1^, ^2^ ^]^ As eCO_2_R catalysts, molecular systems, especially metal complexes,^[^ ^3^, ^4^ ^]^ have been extensively studied along with inorganic^[^ ^5^, ^6^, ^7^ ^]^ and inorganic/molecular hybrid^[^ ^8^ ^]^ systems. Metal‐complex eCO_2_R catalysts including Fe,^[^ ^9^, ^10^, ^11^, ^12^ ^]^ Ni,^[^ ^13^, ^14^, ^15^ ^]^ and Cu^[^ ^16^, ^17^, ^18^, ^19^ ^]^ complexes have been found to exhibit high eCO_2_R performances. Among them, Co complexes are one of the most studied catalysts exhibiting the excellent performance for CO production,^[^ ^20^, ^21^, ^22^, ^23^, ^24^, ^25^, ^26^ ^]^ and their catalytic performances have been enhanced by the molecular designs, mainly focusing on mononuclear complexes. For example, their substrate/product selectivity^[^ ^27^, ^28^ ^]^ and overpotentials^[^ ^29^, ^30^, ^31^ ^]^ have been significantly improved. Considering that eCO_2_R involves the complicated multi‐electron reduction and bond formation/cleavage processes, more flexible electron control and coordination are required to further improve the catalytic performances. For this purpose, the use of dinuclear complexes, which allows the multi‐electron manipulation and multi‐dentate coordination, can be a promising approach.

**Scheme 1:**
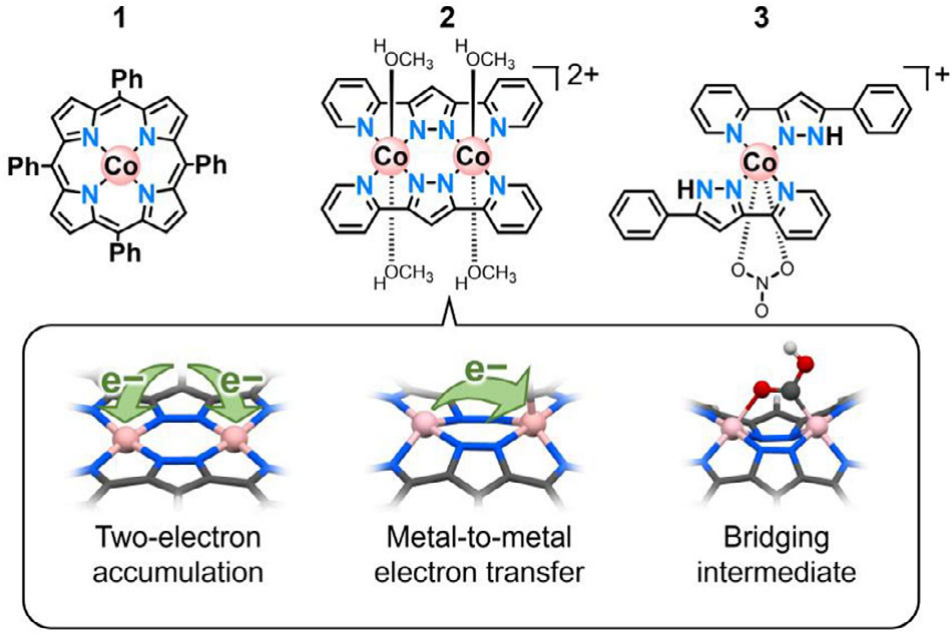
Molecular structures of **1** (top left), **2** (top center), and **3** (top right) and characteristics of **2** (bottom).

Herein, we firstly focused on porphyrin ligand because metal porphyrins are typical electrocatalysts for eCO_2_R.^[^ ^32^ ^]^ The dimerized porphyrin (Fe) catalysts with two porphyrin units bridged by phenylene or phenylene amide group are reported to exhibit efficient eCO_2_R.^[^ ^33^, ^34^, ^35^ ^]^ In this study, aiming to promote the electronic communication between two metal centers, we prompted to locate two metal centers in a close proximity, while maintaining the characteristic properties of metal porphyrins, i.e., the divalent metal ions coordinated by four nitrogen atoms. Inspired by cobalt tetraphenylporphyrin, denoted as **1** (**Scheme** **1** left), because **1** and the related Co‐porphyrins are extensively studied catalysts with high selectivity for CO (best Faradaic efficiency (FE); 99%),^[^ ^36^, ^37^, ^38^, ^39^, ^40^, ^41^, ^42^, ^43^ ^]^ we targeted a dinuclear complex, [Co_2_(bpypz)_2_(CH_3_OH)_4_]^2+^ (**2**, Hbpypz = 3,5‐bis(2‐pyridyl)pyrazole; Scheme 1 center). In addition, we prepared a related mononuclear complex with the ligand bearing a phenyl group instead of a pyridyl group, [Co(NO_3_)(Hphpzpy)_2_]^+^, (**3**, Hphpypz = 3‐phenyl‐5‐(2‐pyridyl)pyrazole; Scheme 1 right).

Through the detailed mechanistic insight gained from experimental and computational studies, we here demonstrate the cooperation of the two metal sites in **2** in eCO_2_R (Scheme 1 bottom), allowing 1) two‐electron accumulation, 2) metal‐to‐metal electron transfer, and 3) a bridging intermediate, which affords the lowered overpotential compared to that of **1**. While there are reports of dinuclear complexes exhibiting some of the aforementioned properties, such as (1) and presumably (3)^[^ ^44^ ^]^ or (2) and (3),^[^ ^45^ ^]^ this study is, to the best of our knowledge, the first case featuring aforementioned three properties.

## 2 Results and Discussion

**Figure 1:**
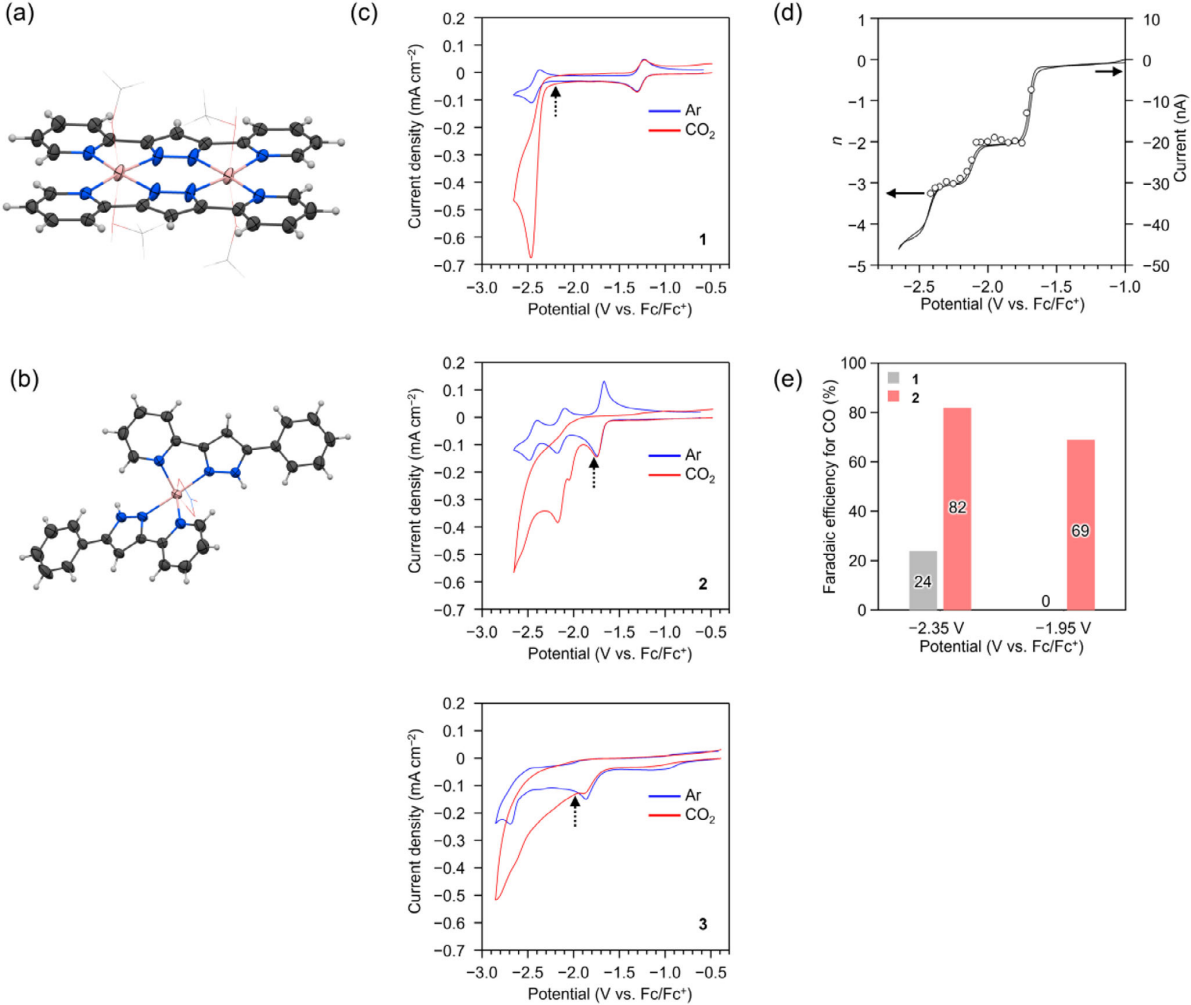
Molecular structures of a) **2** and b) **3** obtained via single‐crystal X‐ray diffraction analysis. Molecules are drawn with ellipsoids set at 50% probability. Color code: C, gray; H, white; N, blue; O, red; Co, pink. CH_3_OH molecules and NO_3_ anion in (a) and (b), respectively, are drawn in wireframe style. c) Cyclic voltammograms of (top) **1** (0.5 mm), (center) **2** (0.5 mm), and **3** (0.5 mm) in DMF with (*n*Bu_4_N)ClO_4_ (0.1 m) under Ar (blue) and CO_2_ (red) at a glassy carbon disk electrode with a diameter of 3.0 mm. Scan rate: 0.05 V s^−1^. Onset potentials of eCO_2_R are shown by arrows. d) Variation of the number of transferred electrons per molecule for **2** as a function of the potential (*n*; open circle, left axis), obtained by the Cottrell plot (see the main text). Cyclic voltammogram of **2** (0.9 mm) in DMF with (*n*Bu_4_N)ClO_4_ (0.2 m) under Ar at a Pt disk electrode with a diameter of 100 µm (solid line, right axis) is also shown. Scan rate: 0.005 V s^−1^. e) Faradaic efficiency for CO during controlled‐potential electrolysis as a function of the applied potential for (left gray bar) **1**, and (right red bar) **2**, respectively.

**2**(BPh_4_)_2_ sample was synthesized by the reaction of cobalt nitrate and the ligand (Hbpypz),^[^ ^46^ ^]^ followed by the anion exchange (see Supporting Information for details). The sample was characterized by elemental analysis, single‐crystal X‐ray diffraction (SCXRD) analysis, and magnetic susceptibility measurement. SCXRD analysis confirmed the flat dinuclear molecular structure with CH_3_OH molecules in labile coordination at apical sites (**Figure** **1**a, see also Figure S1 and Table S1, Supporting Information), which would be advantageous for the coordination of a substrate molecule at multiple centers. The Co─Co distance was 4.13 Å. The temperature dependence of magnetic susceptibility was well fitted by *S* = 3/2 spin pair model, indicating that the two Co ions adopt the Co^II^ state with high‐spin configuration (Figure S3, Supporting Information). **3**(NO_3_) sample was synthesized by the reaction of cobalt nitrate and the ligand (Hphpzpy).^[^ ^47^ ^]^ The sample was characterized by elemental analysis and SCXRD analysis. SCXRD analysis revealed that the Co ion is six‐coordinated by four N atoms of two ligands and two O atoms of a NO_3_^−^ anion (Figure 1b and Figure S2, Supporting Information).

To investigate the redox properties, we performed cyclic voltammetry (CV) with *N*,*N*‐dimethylformamide (DMF) solutions under Ar. On **1**, two reversible waves were observed at ‐1.27 and ‐2.42 V versus ferrocene/ferrocenium (Fc/Fc^+^) (blue curve in Figure 1c top), which were attributed to [L, Co^II^]^0^/[L^−^˙, Co^II^]^−^ and [L^−^˙, Co^II^]^−^/[L^2−^, Co^II^]^2−^ redox couples, respectively,^[^ ^48^ ^]^ where L represents the ligand and Co represents cobalt ion. The assignment was also confirmed by our DFT calculations using the B3LYP functional (see Supporting Information for details). In the following text, all the potentials are listed versus Fc/Fc^+^. On **2**, three reversible waves were observed at ‐1.71, ‐2.14, and ‐2.44 V (blue curve in Figure 1c, center). The first redox wave (‐1.71 V) exhibited significantly greater intensity than the other two, which implies the multi‐electron redox. To determine the number of transferred electrons (*n*), we performed chronoamperometry using a microelectrode.^[^ ^49^, ^50^ ^]^ We obtained *n* from the slope and the intercept in the *I*–*t* ^−1/2^ plot, where *I* and *t* are current and time, respectively (see Figure S4, Supporting Information for details). In Figure 1d, the obtained *n* is plotted against the potential along with the CV curve recorded with a slow scan rate (5 mV s^−1^), clearly showing the redox potentials. The results revealed that the electrons transferred at the first (‐1.71 V) and second (−2.14 V) reductions are two and one, respectively. Furthermore, the zinc analogue ([Zn_2_(bpypz)_2_]^2+^) exhibited no apparent redox waves within the corresponding potential range (Figure S5, Supporting Information), suggesting that the ligand possesses no redox activity. Therefore, the observed three redox waves on **2** were attributed to [L, Co^II^, Co^II^]^2+^/[L, Co^I^, Co^I^]^0^, [L, Co^I^, Co^I^]^0^/[L, Co^I^, Co^0^]^−^, and [L, Co^I^, Co^0^]^−^/[L, Co^0^, Co^0^]^2−^ redox couples, respectively. In contrast to **1** and **2** exhibiting reversible reductions, **3** exhibited two irreversible reductions with the peaks at −1.87 and −2.69 V (Figure 1c bottom).

Next, CV measurements were performed under CO_2_. The CV curves for **1** (red curve in Figure 1c top), **2** (red curve in Figure 1c center), and **3** (red curve in Figure 1c bottom) exhibited deviations from those recorded under Ar (blue curves in Figure 1c), suggesting the eCO_2_R. Notably, the onset potential of **2** (‐1.78 V) was more positive than those of **1** (‐2.20 V) and **3** (‐1.98 V), indicating the superior catalytic activity of **2**. Here, the onset potential was determined to be the potential at which the difference in current density under Ar and CO_2_ reaches 0.01 mA cm^−2^. The onset shift achieved by combining two metal sites (420 mV for **1** vs **2**, 200 mV for **1** vs **3**) is excellent compared to those achieved by the previous systematic studies on Co complexes. For example, a series of tripodal Co complexes bearing ligands with different basicity exhibited the shift of overpotential of 180 mV,^[^ ^51^ ^]^ and Co bis(pyridylmonoimine) complexes bearing ligands with different flexibility exhibited the onset shift of 330 mV.^[^ ^52^ ^]^ The onset potential of **2** is moderate when compared to those of the related nitrogen‐coordinated mononuclear Co complexes (see Table S2, Supporting Information), and hence, further lowering of onset potential may be achieved by combining two metal centers based on these mononuclear complexes. Besides, the maximum turnover frequencies obtained from these CV data based on the foot‐of‐the‐wave analysis^[^ ^53^ ^]^ were 58 and 0.66 s^−1^ for **1** and **2**, respectively (see Supporting Information for details of the analysis). It should be noted that, since the first reduction related to eCO_2_R is irreversible and thus the redox potential was not determined, the foot‐of‐the‐wave analysis cannot be applied to **3**. In the following part, we focus on the reason for the difference in the overpotential afforded by **1** and **2**, because these two catalysts exhibited reversible reductions in Ar, which makes the discussion on the catalytic cycles clear (see below).

To check eCO_2_R products, we performed controlled‐potential electrolysis (CPE) with DMF solutions (see Supporting Information for details). In CPE experiments, 2,2,2‐trifluoroethanol was added in the cathodic chamber as a proton source in addition to either **1** or **2**, and Fc was added in the anodic chamber as a sacrificial reductant. The CV curves recorded with the CPE setup under either Ar or CO_2_ are shown in Figure S9 (Supporting Information). In the CPE experiments performed at ‐2.35 V, which is more negative than the onset potentials of both **1** and **2** observed in CV (**1**; ‐2.20 V, **2**; ‐1.78 V, Figure 1c), CO and H_2_ were generated with both complexes. The FEs for CO were 24% and 82% for **1** and **2**, respectively (Figure 1d left), whereas the FEs for H_2_ were 21% and 2%, respectively. In contrast, in the CPE experiments at ‐1.95 V, which is more negative than the onset potential of **2** but more positive than that of **1** (Figure 1b), CO (FE = 69%) and H_2_ (FE = 6%) were detected for **2**, whereas only H_2_ (FE = 11%) was detected with no eCO_2_R for **1** (Figure 1d right). The reason for the lower sum of FE on **1** can be partly attributed to the electron consumption for the generation of the active one‐electron‐reduced species (see below and Table S3, Supporting Information).^[^ ^36^ ^]^ These results are consistent with the CV measurements and indicate the superior catalytic activity of **2** compared to that of **1**. Besides, to assess catalyst stability during CPE, we monitored the time dependence of current density (Figure S14, Supporting Information) and compared CV curves before and after electrolysis (Figure S15, Supporting Information). A moderate decrease in current density and distortion of the CV curve were observed for CPE with **2** at ‐2.35 V, whereas no significant changes occurred under other conditions, indicating good stability of the catalysts. In addition, for CPE with **2** at ‐1.95 V, ultraviolet–visible (UV–vis) spectra (Figure S16, Supporting Information) showed no significant changes before and after the electrolysis, and scanning electron microscopy–energy dispersive X‐ray spectroscopy (SEM–EDX) analysis of the glassy carbon electrode revealed no apparent Co metal nanoparticle deposition (Figure S17, Supporting Information).

**Figure 2:**
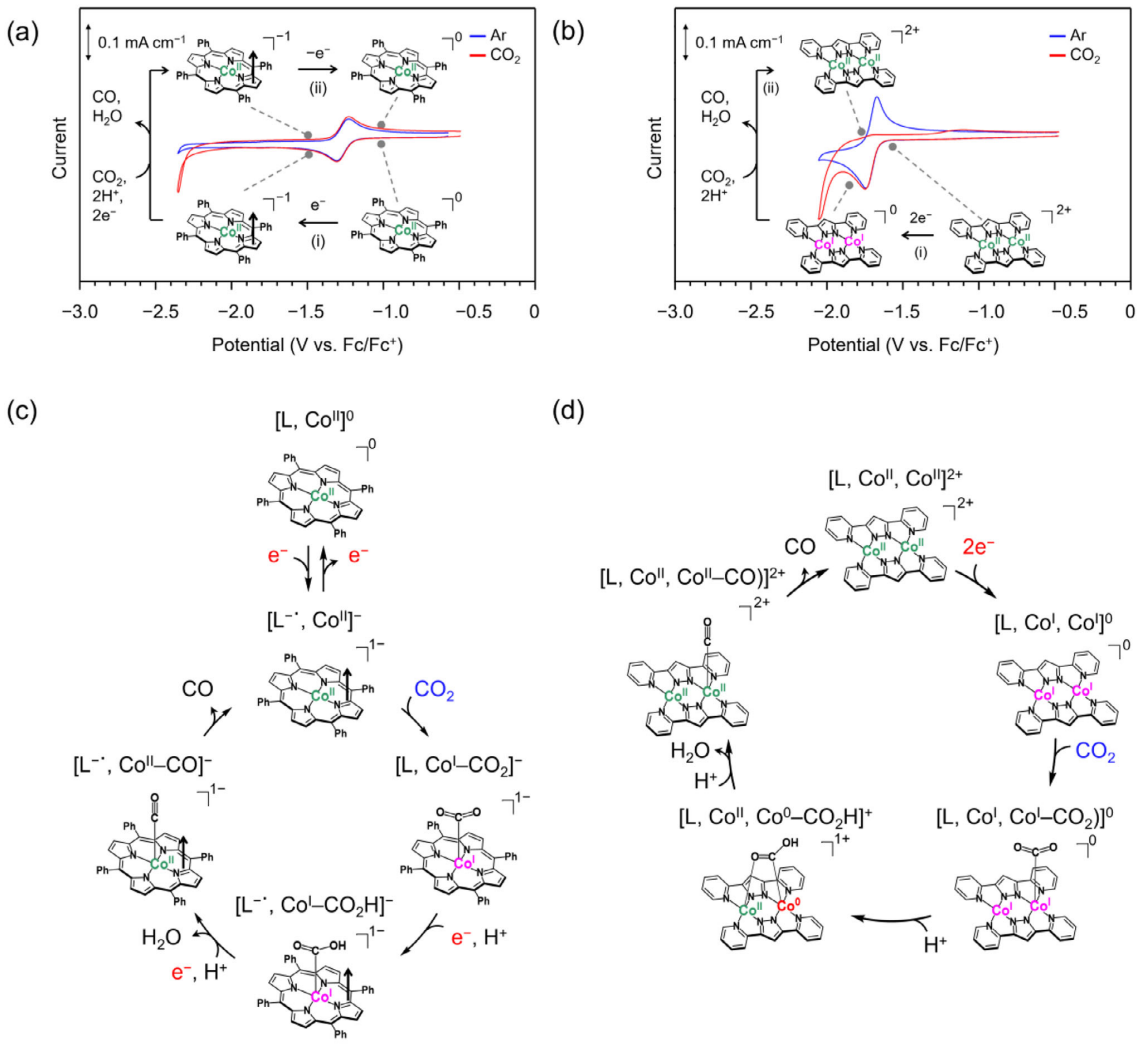
Cyclic voltammograms for a) **1** and b) **2** recorded with the switching potentials slightly negative than the onset potentials recorded under Ar (blue line) and CO_2_ (red line). Schematic molecular structures representing the valence states of Co ions and ligands are shown for selected points. Measurement conditions are the same as Figure 1b. Proposed catalytic cycle of c) **1** and d) **2**. The arrows in the molecular structure indicate the spins.

To consider the catalytic cycles of **1** and **2** at around the onset potential, we performed CV measurements with the switching potential slightly negative than the onset potential (**Figure** **2**a,b). In the cathodic and subsequent anodic scans on **1** under CO_2_, [L, Co^II^]^0^→[L^−^˙, Co^II^]^−^ reduction peak (‐1.31 V; (i) in Figure 2a) and [L^−^˙, Co^II^]^−^→[L, Co^II^]^0^ oxidation peak (‐1.23 V; (ii) in Figure 2a), respectively, were observed. Given that the onset potential for eCO_2_R (‐2.20 V) is more negative than this [L, Co^II^]^0^↔[L^−^˙, Co^II^]^−^ redox potential (‐1.27 V), [L^−^˙, Co^II^]^−^ involves the catalytic cycle, but [L, Co^II^]^0^ is an off‐loop species, i.e., precatalyst. In addition, since the onset potential is more positive than the next [L^−^˙, Co^II^]^−^→[L^2−^, Co^II^]^2−^ reduction potential, the reaction after the generation of [L^−^˙, Co^II^]^−^ is not an electron transfer (denoted as E process^[^ ^12^, ^54^ ^]^) but a chemical reaction; CO_2_ binding (denoted as C process^[^ ^12^, ^54^ ^]^). Notably, this interpretation is consistent with a previous study reporting that [L, Co]^−^ can react with CO_2_ based on the observation by steady state voltammetry.^[^ ^36^ ^]^ Therefore, the reaction sequence from [L^−^˙, Co^II^]^−^ is C→E→E. Proposed catalytic cycle is shown in Figure 2c, where the redox states of the Co ion and ligand obtained from DFT calculation are also shown (see SI). We then discuss **2** (Figure 2b). In the cathodic scan, a reduction peak attributable to [L, Co^II^, Co^II^]^2+^→[L, Co^I^, Co^I^]^0^ ((i) in Figure 2b) was observed at ‐1.74 V, followed by the onset of the catalytic current (≈ ‐1.78 V). Notably, no oxidation peak was observed in the subsequent anodic scan (see (ii) in Figure 2b). This indicates that the electrochemically generated [L, Co^I^, Co^I^]^0^ transforms to [L, Co^II^, Co^II^]^2+^ in the catalytic cycle. Additionally, the position of [L, Co^II^, Co^II^]^2+^→[L, Co^I^, Co^I^]^0^ reduction wave was identical under Ar (Figure 2b blue) and CO_2_ (Figure 2b red), ruling out the possibility of CO_2_ binding by [L, Co^II^, Co^II^]^2+^.^[^ ^55^ ^]^ Therefore, the reaction sequence from [L, Co^II^, Co^II^]^2+^ is two‐electron reduction then CO_2_ binding; 2E→C. Proposed catalytic cycle is shown in Figure 2d. Accordingly, the combined two Co centers change the reaction sequence from C→E→E to 2E→C. Our DFT calculations considering CO_2_‐binding ability in various reduction states also supported these reaction sequences (Figure  S18, Supporting Information). Notably, 2E→C reaction sequence of **2** is the characteristic feature derived from the simultaneous two‐electron reduction, which is rarely observed.^[^ ^44^ ^]^

To discuss why the change in the sequence of the reactions lowers the overpotential, we considered the reaction step that prevents the catalytic cycle at a potential more positive than the onset potential. As for **1**, since the onset potential is located in the potential range between [L, Co^II^]^0^→[L^−^˙, Co^II^]^−^ and [L^−^˙, Co^II^]^−^→[L^2−^, Co^II^]^2−^ reductions, and the cathodic potential sweep triggers the eCO_2_R, the step preventing the catalytic cycle is not the CO_2_ binding but an electron transfer after the CO_2_ binding. It should be noted that the catalytic cycle for **2** does not have this step because of the simultaneous two‐electron reduction prior to the CO_2_ binding. Here, the onset potential for **2** coincides with the reduction wave of [L, Co^II^, Co^II^]^2+^→[L, Co^I^, Co^I^]^0^ (Figure 1b bottom), indicating that the step preventing the catalytic cycle is electrochemical reduction of the catalysts; the subsequent CO_2_ binding and H^+^ additions would have relatively low activation barrier. Thus, the two‐electron accumulation in **2** leads to the circumvention of electron transfer following the CO_2_ binding which prevents the catalytic cycle in **1**, and this is a key for the lowered overpotential of **2**. Therefore, although it is needed to consider the redox potentials of the (pre)catalysts, the use of dinuclear complexes exhibiting preliminary two‐electron reduction can be an effective strategy for invoking the reaction at a relatively positive potential.

**Figure 3:**
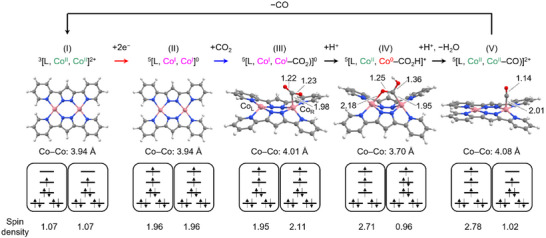
(top) DFT‐optimized structure with the selected bond lengths in Ångström unit, (middle) electronic configuration of *d* orbitals, and (bottom) Mulliken spin densities of Co atoms of the chemical species in the catalytic cycle of **2** at around the onset potential. Color code (top): C, gray; H, white; N, blue; Co, pink; O, red.

Furthermore, we considered the reason for the low activation barrier of the post‐CO_2_‐binding steps on **2** based on DFT calculations. **Figure** **3** shows the optimized molecular structure and the electronic configuration in the catalytic cycle. For each reaction step, the chemical species in the most stable spin state is shown, and the spin multiplicity is indicated as the left superscript. Especially in the first H^+^ addition step ((III)→(IV) in Figure 3), we found two characteristic behaviors, which would contribute to the low activation barrier in the reaction: Metal‐to‐metal electron transfer and the formation of a CO_2_‐derived intermediate bridging across two Co sites. To discuss the metal‐to‐metal electron transfer, we consider the Mulliken spin densities (Figure 3 bottom) of the Co sites. For the catalyst–CO_2_ adduct ((III) in Figure 3), the spin densities of the CO_2_‐binding Co atom (hereafter Co_R_) and the apical‐free Co atom (hereafter Co_L_) are 2.11 and 1.95, respectively, indicating that the electronic configurations of both the two Co sites are high‐spin *d* ^8^ (Co^I^). After the H^+^ addition, the O atom of the HOCO moiety coordinates to the Co_L_, resulting in the HOCO‐bridging intermediate ((IV) in Figure 3). In the reaction from (III) to (IV), the spin densities of Co_R_ and Co_L_ change to 0.96 and 2.71, respectively. At the same time, the charges of Co_R_ and Co_L_ change from −0.64 and 0.34 to −1.47 and 0.79, respectively (Tables S5 and S6, Supporting Information). These results indicate the electron transfer from Co_L_ to Co_R_, and the electronic configurations of Co_L_ and Co_R_ change to d^9^ (Co^0^) and high‐spin d^7^ (Co^II^), respectively. Apparently, this electron transfer is a unique cooperative behavior afforded by the combined two Co centers. In addition, the realization of the intermediate species with a Co─C─O─Co‐type bridging ((IV) in Figure 3) is also a significant cooperative coordination behavior of two metal centers. It should be noted that the calculated Co─Co distance in initial [Co_2_(bpypz)_2_]^2+^ state ((I) in Figure 3) is 3.94 Å, whereas that in HOCO‐bridged intermediate ((IV) in Figure 3) is 3.70 Å. This remarkable shortening of the Co─Co distance following the attachment of HOCO moiety is the result of the significant distortion of the entire complex structure, as illustrated in Figure 3. This structural flexibility would be one of the characteristic properties of **2**. It is valuable to point out the similarity between **2** and the natural CO_2_/CO‐converting enzyme CO‐dehydrogenase (CODH) with multimetallic active sites; the CO_2_‐binding intermediate of CODH has Ni─C─O─Fe‐type bridging.^[^ ^56^ ^]^ With regard to the effect of the spin state on the catalytic process, it should be noted that the spin state of the Co_L_ changes from high‐spin state to low‐spin state on the CO‐release step (Figure 3 (V) → (I)). If the complex binds CO with Co_L_ in the low‐spin state, the Gibbs energy of the system would be 8.3 kcal mol^−1^ higher than that with Co_L_ in the high‐spin state (see Table S7, Supporting Information) (charge, multiplicity) = (2,3) for Co_L_ in low‐spin state and (2,5) for Co_L_ in high‐spin state), which would hinder the progress of the catalytic cycle. Therefore, the capability to adopt the two spin states of **2**, i.e., high‐spin state and low‐spin state, would contribute to efficient catalytic behavior.

## 3 Conclusion

In conclusion, we demonstrated that the cooperation of two metal centers of a dinuclear complex in eCO_2_R. The combined two metal centers afforded simultaneous two‐electron reduction, which leads to the circumvention of the reaction step hindering the catalytic cycle. Moreover, characteristic metal‐to‐metal electron transfer and bridging intermediate were found, which would contribute to the low activation barrier. Thus, the use of the unique flexibility of the electron manipulation and adaptive coordination ability of dinculear complexes can be an effective approach to achieve efficient eCO_2_R performance. We believe that these findings would contribute not only to eCO_2_R research but also to research on other catalytic reactions.

[CCDC 2403443 and 2480960 contain the supplementary crystallographic data for this paper. These data can be obtained free of charge from The Cambridge Crystallographic Data Centre via www.ccdc.cam.ac.uk/data_request/cif.]

## Conflict of Interest

The authors declare no conflict of interest.

## Supporting information



Supporting Information

Supporting DataFile

## Data Availability

The data that support the findings of this study are available from the corresponding author upon reasonable request.
