# Supplementary material for: Cooperation of Two Metal Centers in a CO2 Electroreduction Catalyst: Flexible Electron Manipulation and Adaptive Coordination on a Dinuclear Cobalt Complex
Source: Adv Sci (Weinh). 2025 Oct 14;12(46):e08361. doi: 10.1002/advs.202508361 (PMC12697882; doi:10.1002/advs.202508361)
Supplement: Supplementary file 1 — Supporting Information [file ADVS-12-e08361-s002.pdf]

Supporting Information for

Cooperation of Two Metal Centers in a CO_2 Electroreduction Catalyst: Flexible Electron Manipulation and Adaptive Coordination in a Dinuclear Cobalt Complex

Yunyi Pan,[a] Masaki Donoshita,*[a][b] Yohei Kametani,[b][c] Yoshihito Shiota,[b][c] Shu-Qi Wu,[a][b] Osamu Sato,[a][b] Miho Yamauchi*[a][b][d][e][f]

[a] Department of Chemistry, Graduate School of Science, Kyushu University, Motooka 744, Nishi-ku, Fukuoka 819-0395, Japan

[b] Institute for Materials Chemistry and Engineering (IMCE), Kyushu University, Motooka 744, Nishi-ku, Fukuoka 819-0395, Japan

[c] Department of Applied Chemistry, Graduate School of Engineering, Kyushu University Motooka 744, Nishi-ku, Fukuoka 819-0395, Japan

[d] International Institute for Carbon-Neutral Energy Research (WPI-I^{2}CNER), Kyushu University, Motooka 744, Nishi-ku, Fukuoka 819-0395, Japan

[e] Research Center for Negative Emissions Technologies (K-NETs), Kyushu University, Motooka 744, Nishi-ku, Fukuoka 819-0395, Japan

[f] Advanced Institute for Materials Research (WPI-AIMR), Tohoku University, 2-1-1 Katahira, Aoba-ku, Sendai 980-8577, Japan

Y. Pan and M. Donoshita contributed equally to this work.

*E-mail:

donoshita@ms.ifoc.kyushu-u.ac.jp
yamauchi@ms.ifoc.kyushu-u.ac.jp

Contents

S1. Experimental methods

S2. Single-crystal X-ray diffraction analysis

S3. Magnetic behavior of **2**$(BPh_4)_2$

S4. Cottrell plot for **2**$(BPh_4)_2$

S5. CV of $[Zn_2(bpypz)_2](NO_3)_2 \cdot CH_3OH$

S6. Determination of turnover frequencies by foot-of-the-wave analysis

S7. Comparison of onset potentials with related compounds

S8. Controlled-potential electrolysis

S9. DFT calculations

S10. References

S1. Experimental methods

S1.1 Materials

Cobalt tetraphenylporphyrin (**1**, Tokyo Chemical Industry), $Co(NO_3)_2 \cdot 6H_2O$ (FUJIFILM Wako Chemical), 3,5-bis(2'-pyridyl)pyrazole (Hbpypz, Tokyo Chemical Industry), MeOH (FUJIFILM Wako Chemical), $NaBPh_4$ (FUJIFILM Wako Chemical), tetrahydrofuran (THF, FUJIFILM Wako Chemical), anhydrous THF (FUJIFILM Wako Chemical), EtOH (FUJIFILM Wako Chemical), $Zn(NO_3)_2 \cdot 6H_2O$ (FUJIFILM Wako Chemical), *N*,*N*-dimethylformamide (DMF, FUJIFILM Wako Chemical), tetrabutylammonium perchlorate ($TBAClO_4$, FUJIFILM Wako Chemical), ferrocene (FUJIFILM Wako Chemical), and 2,2,2-trifluoroethanol (FUJIFILM Wako Chemical) were used without further purification.

S1.2 Elemental analysis

Elemental analysis was performed for C, H and N by the Service Center of the Elementary Analysis of Organic Compounds, Faculty of Science, Kyushu University.

S1.3 Thermogravimetry

Thermogravimetry analyses were performed using Bruker TG–DTA2010SA instrument at 2 K min^{-1} in a N_2 atmosphere.

S1.4 Synthesis of $[Co_2(bpypz)_2(CH_3OH)_4](BPh_4)_2$

$[Co_2(bpypz)_2(CH_3OH)_4](BPh_4)_2$ (hereafter **2**$(BPh_4)_2$) was synthesized via a two-step synthesis; i.e., the synthesis of $[Co_2(bpypz)_2](NO_3)_2 \cdot 2H_2O$ according to a literature[S1] and the following anion exchange.

Firstly, to a MeOH solution (50 mL) containing $Co(NO_3)_2 \cdot 6H_2O$ (1.31 g, 4.5 mmol), was added a MeOH solution (50 mL) containing 3,5-di(2-pyridyl)pyrazole (Hbpypz; 500 mg, 2.3 mmol). The resulting solution was stirred under aerobic conditions at room temperature for 24 h, and the formed orange precipitate was filtered and dried *in vacuo*, yielding orange powders of $[Co_2(bpypz)_2](NO_3)_2 \cdot 2H_2O$ (294 mg, 0.41 mmol) with the yield of 36%.

Elemental analysis:

calcd (%) for $[Co_2(bpypz)_2](NO_3)_2·2H_2O$: C 43.35, H 3.08, N 19.44.

found (%) : C 43.82, H 3.09, N 19.56.

Next, to an aqueous solution (100 mL) containing $[Co_2(bpypz)_2](NO_3)_2·2H_2O$ (108 mg, 0.15 mmol), was added $NaBPh_4$ (205 mg, 0.60 mmol). After stirring for 15 min, the formed precipitate was filtered and washed with H_2O (4×5 mL). The obtained pale-orange solid was recrystallized from a mixed solvent of MeOH and THF (5:1) and then dried *in vacuo*, yielding orange solids of $[Co_2(bpypz)_2(CH_3OH)_4](BPh_4)_2$ (91.0 mg, 0.069 mmol) with the yield of 46%. Notably, fresh crystals obtained after the recrystallization had extra MeOH as a crystal solvent, namely, whose formula is $[Co_2(bpypz)_2(CH_3OH)_4](BPh_4)_2·(CH_3OH)$ (hereafter **2**$(BPh_4)_2·CH_3OH$), and such fresh sample was used for the single-crystal X-ray diffraction analysis (see below).

Elemental analysis:

calcd (%) for $[Co_2(bpypz)_2(CH_3OH)_4](BPh_4)_2$: C 70.60, H 5.62, N 8.44.

found (%) : C 70.31, H 5.61, N 8.42.

S1.5 Synthesis of $[Co(NO_3)(Hphpzpy)_2]NO_3·0.5H_2O$

The ligand, 3-phenyl-5-(2-pyridyl)pyrazole (Hphpzpy), was synthesized according to the literature[S2] with a slight modification; namely ethyl picolinate was used instead of methyl picolinate.

Elemental analysis:

calcd (%) for $C_{14}H_{11}N_3$: C 76.00, H 5.01, N 18.99.

found (%) : C 75.88, H 4.95, N 19.00.

$[Co(NO_3)(Hphpzpy)_2]NO_3·0.5H_2O$ (hereafter **3**$(NO_3)·0.5H_2O$) was synthesized according to a literature.[S2] The reaction was carried out under Ar atmosphere. To a solution of $Co(NO_3)_2·6H_2O$ (30.5 mg, 0.11 mmol) in anhydrous THF (100 mL), was added Hphpzpy (50.0 mg, 0.23 mmol) at room temperature. After stirring for 28 h, pale pink precipitate was filtered and dried *in vacuo*, yielding pale pink powders of $[Co(NO_3)(Hphpzpy)_2]NO_3·0.5H_2O$ (61.6 mg, 0.097 mmol) with the yield of 88%. Although the sample containing one molecule of H_2O per formula was reported in the literature,[S2] we obtained the sample containing half a molecule of H_2O per formula, which was confirmed by elemental analysis. Suitable crystals for X-ray diffraction

experiments were obtained by recrystallization from a mixed solvent of MeOH and EtOH (1:1), which has no H_2O (hereafter **3**(NO_3)).

Elemental analysis:

calcd (%) for [Co(NO_3)(Hphpzpy)$_2$]NO_3·0.5H_2O : C 53.45, H 3.71, N 17.35.

found (%) : C 53.01, H 3.65, N 17.66.

S1.6 Synthesis of [Zn_2(bpypz)$_2$](NO_3)$_2$·CH_3OH[S1]

To a MeOH solution (25 mL) containing Zn(NO_3)$_2$·6H_2O (669 mg, 2.3 mmol) was added a MeOH solution (25 mL) containing 3,5-di(2-pyridyl)pyrazole (Hbpypz; 250 mg, 1.1 mmol). The resulting solution was stirred under aerobic conditions at room temperature for 24 h, and the formed precipitate was filtered and dried *in vacuo*, yielding white powders of [Zn_2(bpypz)$_2$](NO_3)$_2$·CH_3OH (hereafter **Zn2**(NO_3)$_2$·CH_3OH; 186 mg, 0.26 mmol) with the yield of 45%. Although the tetrahydrate sample was reported in the literature,[S1] we obtained the sample containing MeOH, which was confirmed by thermogravimetry (found weight loss; 4.67% at approximately 100–160 °C vs. calcd. weight loss; 4.40%) and elemental analysis.

Elemental analysis:

calcd (%) for [Zn_2(bpypz)$_2$](NO_3)$_2$·MeOH : C 44.47, H 3.04, N 19.21.

found (%) : C 44.52, H 2.96, N 19.16.

S1.7 Single-crystal X-ray diffraction (SCXRD) analysis

For the SCXRD analysis, the diffraction data were collected using a RIGAKU XtaLAB Synergy-R/DW diffractometer equipped with a RIGAKU Hypix-6000HE detector. For complex **2**, a fresh crystal obtained after the recrystallization from a mixed solvent of MeOH and THF (5:1), whose formula is [Co_2(bpypz)$_2$(CH_3OH)$_4$](BPh_4)$_2$·(CH_3OH), was investigated (see above). For complex **3**, a crystal obtained after the recrystallization from a mixed solvent of MeOH and EtOH (1:1), whose formula is [Co(NO_3)(Hphpzpy)$_2$]NO_3, was investigated (see above). The structures were solved by direct methods (SHELXT)[S3] and refined by full-matrix least-squares refinement on F^2 (SHELXL)[S4] using the Olex2 software package.[S5] All non-hydrogen atoms were refined anisotropically. The positional parameters of the hydrogen atoms were calculated using the sp^2 or sp^3 configuration of the bonding atoms.

In the refinement procedures, isotropic atomic displacement parameters with magnitudes of 1.2-fold to those of the equivalent isotropic atomic displacement parameters of the bonding atoms were applied for the hydrogen atoms.

S1.8 Magnetic susceptibility

Magnetic susceptibility measurements were performed using a quantum design MPMS-5S superconducting quantum interference device (SQUID) magnetometer. Polycrystalline samples were loaded into a gelatin capsule, which was fixed on a sample rod with a plastic straw. Measurements were conducted with dc 2000 Oe in the temperature range of 2–300 K. The contribution of core diamagnetism (-6.093×10^{-4} emu mol^{-1} for **2**(BPh_4)$_2$) was subtracted using Pascal's constant.

S1.9 Electrochemical experiments

Electrochemical experiments were performed using a Princeton Applied Research PARSTAT MC CHS08A or PARSTAT MC-200 at room temperature. Ferrocene was used as a standard, and all potentials are referenced to the ferrocene/ferrocenium (Fc/Fc^+) couple at 0 V.

S1.9.1 Cyclic voltammetry (CV)

CV measurements were performed using a one-compartment cell with a three-electrode configuration, which consisted of a glassy carbon disk (diameter 3.0 mm, from BAS) as the working electrode, Pt coil as the counter electrode, and Ag/Ag^+ (Ag/0.01 M $AgNO_3$, from BAS) as the reference electrode.

S1.9.2 Chronoamperometry (CA) and Cottrell plot

CA measurements using a microelectrode were performed using a one-compartment cell with a three-electrode configuration. A Pt disk electrode with a diameter of 100 μm (BAS) was used as a working electrode. The electrode surface was polished with 0.05 μm alumina before each run. A Pt coil and Ag/Ag^+ (Ag/0.01 M $AgNO_3$, from BAS) were used as the counter and reference electrodes, respectively. Ar gas was bubbled through the solution for 30 min after the cell assembly, and all the runs were performed under Ar atmosphere.

The sample solution was prepared by dissolving $TBAClO_4$ (0.68 g, 0.2 M) and **2**$(BPh_4)_2$ (11.9 mg, 0.9 mM) into 10 mL DMF. Additionally, a blank solution was prepared by dissolving only $TBAClO_4$ (0.68 g, 0.2 M) into 10 mL DMF for background-subtraction.

CA measurements were carried out within the potential range of approximately −1.6 to −2.4 V vs. Fc/Fc^+. For each potential, the current was recorded every 0.01 s for 3 s after the potential was stepped from the open-circuit potential. The current (I) obtained for the blank solution was subtracted from that for the sample solution. The background-subtracted I was plotted against $t^{-1/2}$ (Cottrell plot), and the slope (s) and the intercept (p) for the linear regression line for the data points within 1.1 $s^{-1/2} < t^{-1/2} < 1.4$ $s^{-1/2}$ were calculated (Figure S4). The numbers of the transferred electrons were determined by the equation

$$n = s^2/pFa^3c^*$$

where F is the Faradaic constant, a is the radius of the electrode, and c^* is the concentration of **2**.[S6] It should be noted that we used 77 μm for a in the above equation to obtain plausible n, although a measured using an optical microscope was ~50 μm. The use of such a larger radius might be reasonable considering the microscopic surface roughness of the electrode.

Additionally, to clearly investigate the redox potentials of the sample, a CV curve was recorded using the same setup and solution with a scan rate of 0.005 V s^{-1}.

S1.9.3 Controlled-potential electrolysis (CPE)

CPE was performed in a two-compartment H-type cell separated by an anion exchange membrane Selemion DSVN (Figure S8). In the cathode compartment, a glassy carbon plate as the working electrode and an Ag/Ag^+ (Ag/0.01 M $AgNO_3$) reference electrode were immersed into a DMF solution (16 mL) containing 0.5 mM catalyst, 0.1 M $TBAClO_4$, and 10% 2,2,2-trifluoroethanol. In the anode compartment, a Pt mesh as the counter electrode was immersed into the DMF solution (16 mL) containing 0.1 M $TBAClO_4$ and 40 mM ferrocene as a sacrificial reductant. Prior to electrolysis, the solution of both compartments was babbled with firstly Ar and secondly CO_2 for more than 30 min each. The CV curves under either Ar or CO_2 are recorded before CPE (Figure S9). During CPE measurements, catholyte was constantly bubbled with CO_2 (~16–17 mL min^{-1}). After 60 min electrolysis, gas products were

analyzed by on-line gas chromatography (Inficon, Micro GC Fusion) with a Molsieve 5A column equipped with a thermal conductivity detector to quantify the products, H_2 (Figure S10) and CO (Figure S11). After 60 min electrolysis, catholyte was analyzed by high-performance liquid chromatography (HPLC, Shimadzu, LC-20AD) with a Shodex RSpak KC-811 column equipped with a refractive-index detector to quantify HCOOH, another two-electron-reduced product, although no apparent signal was observed (Figures S12, S13). Faradaic efficiencies shown in the main text are the average of Faradaic efficiencies obtained by two experiments (Table S3).

S1.10 UV-vis spectroscopy

UV−vis spectra were recorded on a SHIMADZU UV-2600i spectrophotometer at room temperature.

S1.11 SEM–EDX analysis

Scanning electron microscopy–energy dispersive X-ray spectroscopy (SEM–EDX) analysis was performed with JEOL JSM-7900F microscope equipped with Oxford instruments X-Max.

S1.12 DFT Calculation

All optimized structures were obtained using the spin-unrestricted B3LYP functional[S7] together with the Grimme-D3 dispersion correction[S8] and the solvent effect in *N'N*-dimethylformamide based on the polarizable continuum model,[S9] as implemented in the Gaussian 16 package.[S10] The (15s11p6d) primitive set of Wachters−Hay supplemented with one polarization *f*-function ($\alpha = 1.17$ for Co)[S11] was used for the Co atoms, and 6-31+G** basis set[S12] for all the other atoms (H, C, N, and O). After structural optimization, the vibration frequencies were systematically computed to ensure that the potential energy surface on each optimized geometry corresponded to a local minimum with no imaginary frequencies. Gibbs free energy G was obtained assuming 298.15 K and 1.00 atm. Because the complexes can adopt various spin states, several states with different spin multiplicities were evaluated (Tables S4–S11, Figures S19–S28). The state with smallest G was considered as the plausible state.

S2. Single-crystal X-ray diffraction analysis

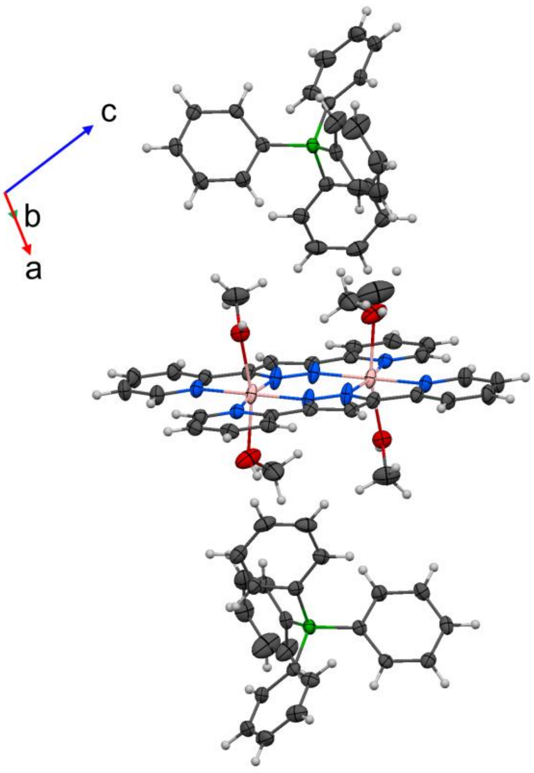

Figure S1. Molecular structures in **2**$(BPh_4)_2{\cdot}CH_3OH$ obtained by SCXRD. Molecules are drawn with ellipsoids set at 50% probability. The non-coordinating CH_3OH molecule is omitted. Color code: C, gray; H, white; N, blue; Co, pink; O, red; B, green.

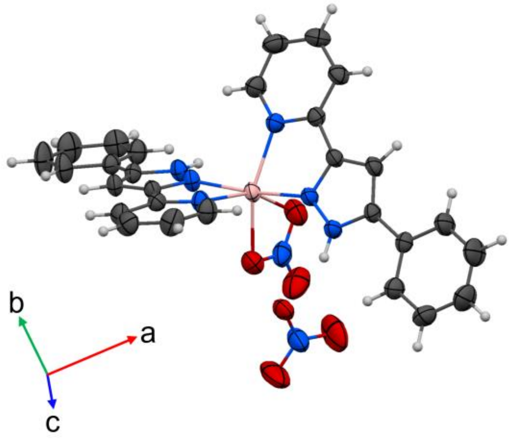

Figure S2. Molecular structures in **3**NO_3 obtained by SCXRD. Molecules are drawn with ellipsoids set at 50% probability. Color code: C, gray; H, white; N, blue; Co, pink; O, red.

Table S1. Cell parameters and crystallographic information for **2**$(BPh_4)_2·CH_3OH$ and **3**(NO_3)

	**2**$(BPh_4)_2·CH_3OH$	**3**(NO_3)
Formula	$C_{79}H_{78}N_8O_5B_2Co_2$	$C_{28}H_{22}N_8O_6Co$
Formula weight	1358.97	625.46
Temperature (K)	100	301
Wavelength (Å)	1.54184	0.71073
Crystal system	Triclinic	Monoclinic
Space group	$P-1$	$C2$
Color	Orange	Orange
a (Å)	11.4357(4)	14.2040(4)
b (Å)	12.8027(6)	12.1594(3)
c (Å)	13.5356(5)	7.8736(2)
α (°)	101.316(4)	90
β (°)	95.477(3)	95.333(3)
γ (°)	114.981(4)	90
V (Å^3)	1725.59(13)	1353.99(7)
Z	1	2
Calcd density (g cm^{-3})	1.308	1.534
μ (mm^{-1})	4.227	0.694
F(000)	712	642
Crystal size (mm^3)	0.236×0.111×0.084	0.096×0.041×0.032
Total reflections	21715	14613
Unique reflections	6917	3115
Parameters used	465	198
R_{int}	0.0333	0.0267
Goodness-of-fit	1.039	1.052
R_1 [$I > 2\sigma(I)$]	0.0397	0.0276
wR_2 (all reflections)	0.1001	0.0654
max, min $\Delta\rho$ (e Å^{-3})	0.47, −0.33	0.43, −0.13
CCDC deposition no.	2403443	2480960

S3. Magnetic behavior of $\mathbf{2}(BPh_4)_2$

Magnetic susceptibility χ of $\mathbf{2}(BPh_4)_2$ was 1.52×10^{-2} emu mol^{-1} at 300 K and exhibited a broad maximum at around 20 K with decreasing temperature (Figure S3a). This temperature dependence was well fitted by $S = 3/2$ antiferromagnetically coupled spin pair model ($H = -2J\boldsymbol{S}_A\cdot\boldsymbol{S}_B$),[S13] which indicates that the two Co ions adopt high-spin d^7 configuration (Co^{II}). The fitting function is

$$\chi = \frac{2Ng^2{\mu_B}^2}{k_BT}\frac{14 + 5\exp\left(-\frac{6J}{k_BT}\right) + \exp\left(-\frac{10J}{k_BT}\right)}{7 + 5\exp\left(-\frac{6J}{k_BT}\right) + 3\exp\left(-\frac{10J}{k_BT}\right) + \exp\left(-\frac{12J}{k_BT}\right)}$$

where N is Avogadro's number, g is g-factor, μ_B is Bohr magneton, J is intramolecular coupling between two Co sites, k_B is Boltzmann's constant. Obtained parameters are $J/k_B = -6.79(2)$ K and $g = 2.309(2)$. Notably, the obtained high-spin state is not consistent with the result of our DFT calculation suggesting a low-spin ground state. However, the discrepancy is reasonable considering the difference in the environment of the complex in each experiment, i.e., the expected apical coordination of MeOH in the SQUID experiment and the enclosure by the solvent in the DFT calculation.

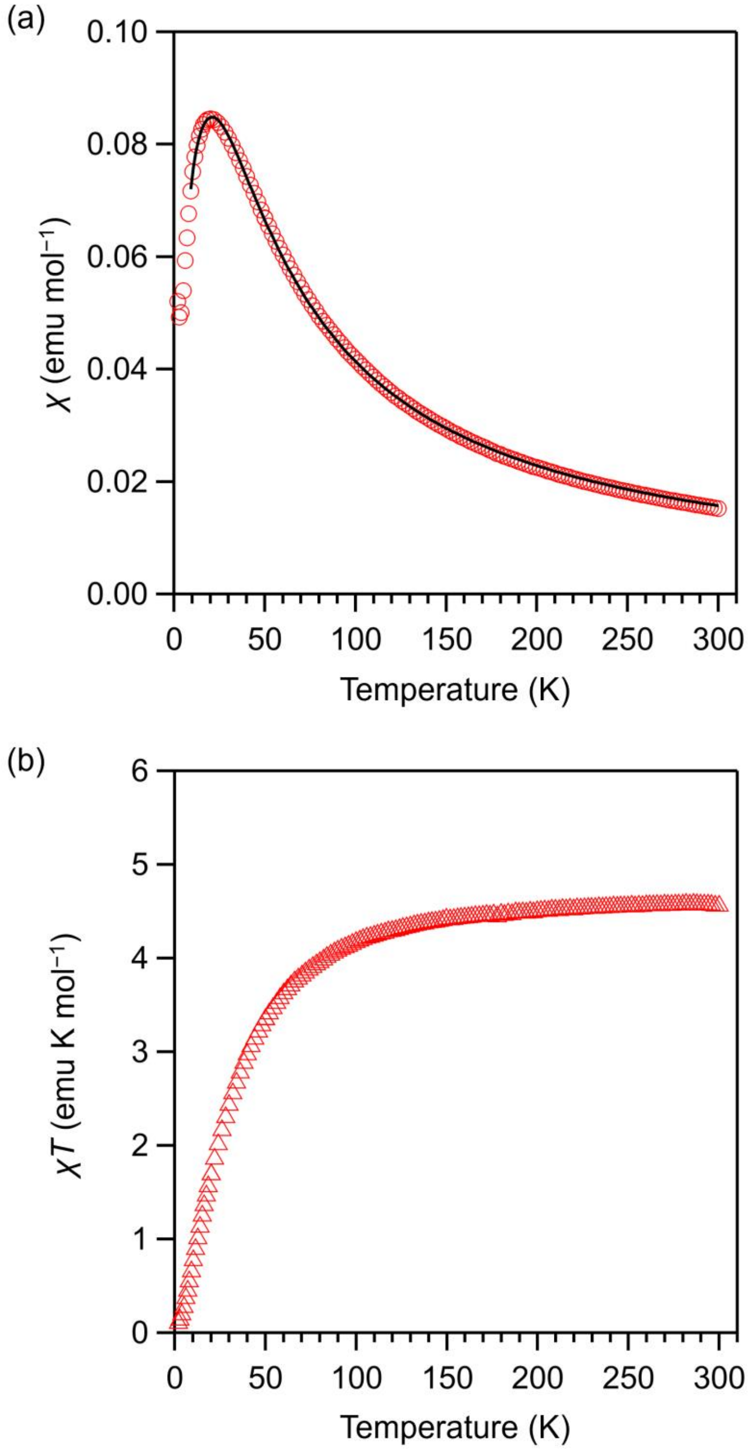

Figure S3. (a) Temperature dependence of magnetic susceptibility χ for **2**(BPh_4)$_2$. Open circle: measured data. Solid line: fitting line of the S = 3/2 antiferromagnetically coupled spin pair model (see the above text). (b) Temperature dependence of χT for **2**(BPh_4)$_2$.

S4. Cottrell plot for 2$(BPh_4)_2$

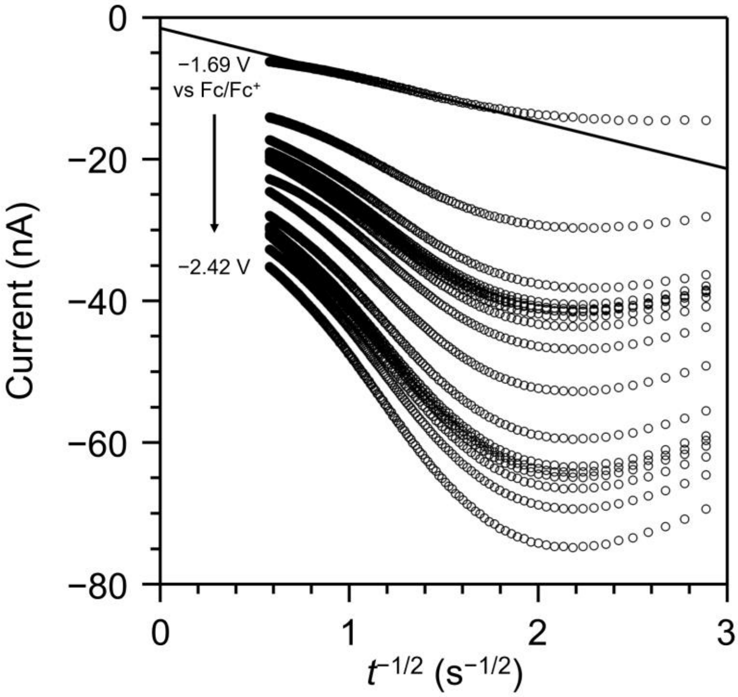

Figure S4. Cottrell plots of the chronoamperograms at the Pt disk electrode in 0.1 mm diameter in the DMF solution of 0.9 mM **2**$(BPh_4)_2$ + 0.2 M $TBAClO_4$ when potential was stepped from open-circuit potential to −1.69–−2.42 V vs Fc/Fc^+. Open circle: measured data. Solid line: fitting line for the data taken at −1.69 V vs Fc/Fc^+ (see Section S1).

S5. CV of Zn2$(NO_3)_2$·CH_3OH

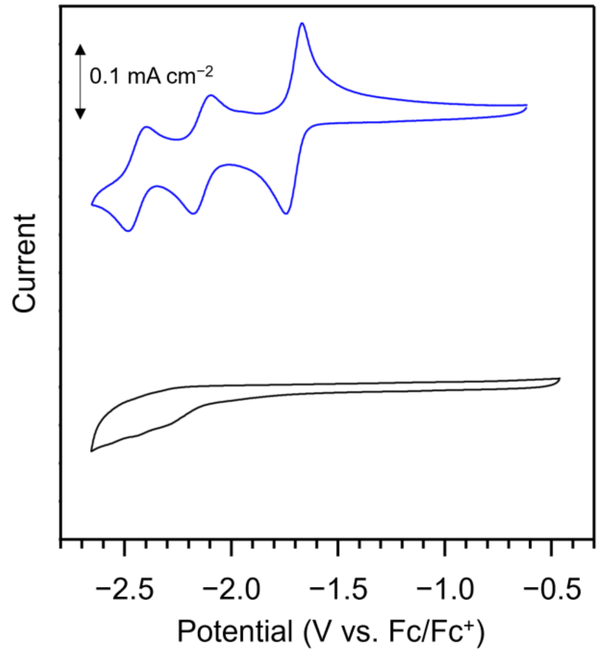

Figure S5. Cyclic voltammograms of (blue) **Co2**$(BPh_4)_2$ (0.5 mM) and (black) **Zn2**$(NO_3)_2$·CH_3OH (0.5 mM) in DMF with $TBAClO_4$ (0.1 M) under Ar at a glassy carbon electrode with a diameter of 3.0 mm. Scan rate: 0.05 V s^{-1}.

S6. Determination of turnover frequency by foot-of-the-wave analysis

We determined the maximum turnover frequencies (TOF_{max}s) for **1** and **2**, based on the foot-of-the-wave analysis[S14, S15] on cyclic voltammetry data (Figure 1c).

The TOF_{max} (= k_{obs}) was obtained according to the following equation:

$$\frac{i}{i_p^0} = \frac{2.24\sqrt{\frac{k_{\text{obs}}}{fv}}}{1 + \exp\{f(E - E_{cat}^0)\}}$$

where i is the current in the presence of CO_2, i_p^0 is the peak current of the catalyst in the absence of CO_2, k_{obs} is the observed rate constant, $f = F/RT$ (F: Faraday constant, R: gas constant, T: temperature, 298.15 K), v is the scan rate (0.05 V s^{-1}), E is the potential, and E_{cat}^0 is the redox potential of the catalyst in the absence of CO_2.

E_{cat}^0 was determined from the CV curve under Ar (−2.416 V for **1** and −1.706 V for **2**). i_p^0 was determined by taking the difference between peak cathodic current and baseline current (at −1.75 V for **1** and −1.45 V for **2**). For i, we also performed the subtraction of the baseline current. From the linear fitting of (i/i_p^0) vs $1/[1+\exp\{f(E-E_{cat}^0)\}]$, we obtained $2.24(k_{obs}/fv)^{1/2}$ as the slope, and hence $k_{obs} = TOF_{max}$ (Figure S6). The obtained TOF_{max}s were 58 s^{-1} and 0.66 s^{-1} for **1** and **2**, respectively.

It should be noted that E_{cat}^0 for **1** was determined for the second redox, i.e., $[L^{-\bullet}, Co^{II}]^-/[L^{2-}, Co^{II}]^{2-}$, for this analysis, although we consider that the eCO_2R proceeds even with $[L^{-\bullet}, Co^{II}]^-$ species (see main text).

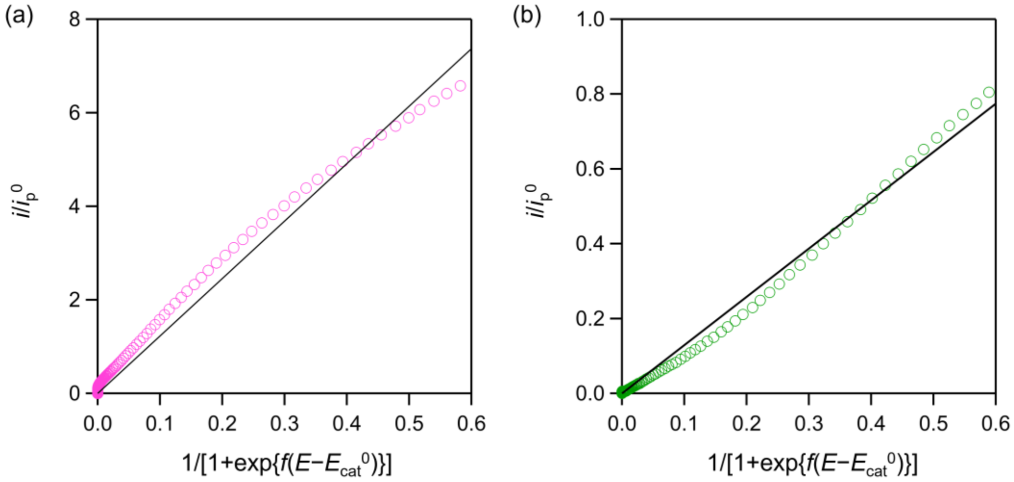

Figure S6. Foot-of-the-wave analysis plot for (a) **1** and (b) **2**. Colored circles are experimental data and black line is the fitting curve.

S7. Comparison of onset potentials with related compounds

Onset potentials for eCO_2R of **1**, **2**, **3**, and those of related nitrogen-coordinated cobalt complexes investigated in organic solvents are summarized in Table S2 (refer to each reference for the method for determining the onset potential). The molecular structures of listed complexes are summarized in Figure S7.

Table S2. Comparison of catalytic performances of **1**, **2**, and **3** with related compounds.

No.	Catalyst	Solvent	Onset potential / V vs. Fc/Fc^+	Ref.
1	**1**	0.1 M (nBu$_4$N)ClO$_4$ in DMF	−2.20	This work
2	**2**	0.1 M (nBu$_4$N)ClO$_4$ in DMF	−1.78	This work
3	**3**	0.1 M (nBu$_4$N)ClO$_4$ in DMF	−1.98	This work
4	**4**	0.1 M (nBu$_4$N)PF$_6$ in DMF	−1.86	S16
5	**5**	0.1 M (nBu$_4$N)PF$_6$ in DMF	−1.63	S16
6	**6**	0.1 M (nBu$_4$N)PF$_6$ in DMF	−1.59	S16
7	**7**	0.1 M (nBu$_4$N)PF$_6$ in DMF	−1.53	S16
8	**8**	2.0% water + 0.1 M (nBu$_4$N)ClO$_4$ in DMF	−1.9	S17
9	**9**	0.1 M (nBu$_4$N)ClO$_4$ in DMF/H_2O (95:5, v:v)	−1.80	S18
10	**10**	3 M water + 0.1 M (nBu$_4$N)PF$_6$ in DMF	−1.84	S19

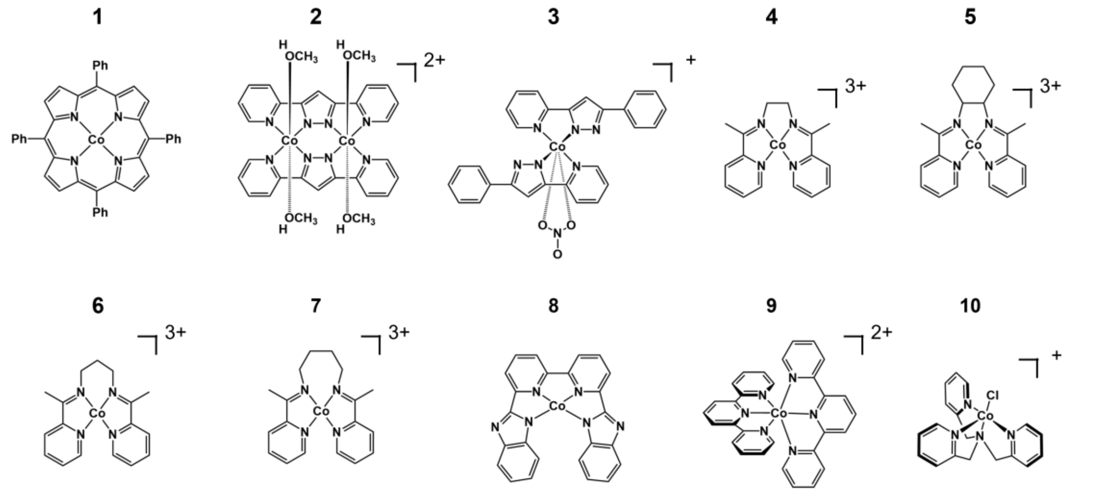

Figure S7. Molecular structures for catalysts listed in Table S2.

S8. Controlled-potential electrolysis

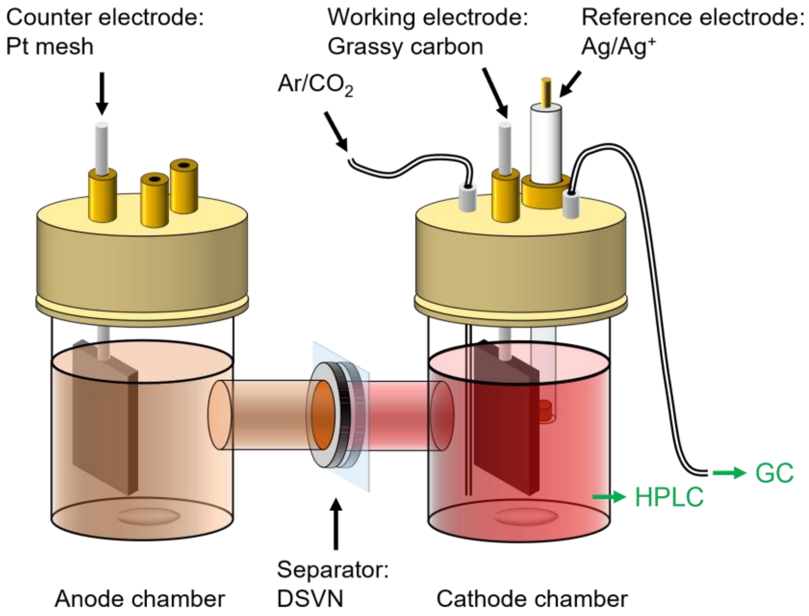

Figure S8. Setup of two-component H-type cell for CPE measurements.

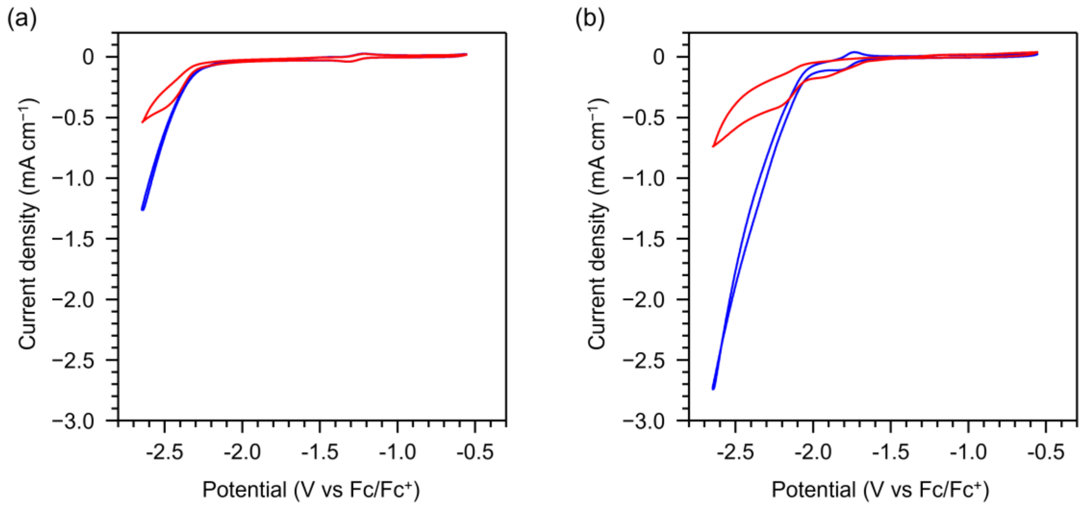

Figure S9. CV curves taken before CPE for **1** (a) and **2** (b) under Ar (blue) and CO_2 (red).

Table S3. Summary of the CPE experiments.

Entry No.	Catalyst	Potential / V vs Fc/Fc$^+$	Total charge / C	Faradaic efficiency / %			
				CO	H_2	HCOOH	FE_{red}[a]
1	**1**	−1.95	−1.05	n.d.[b]	11	n.d.	73
2	**1**	−1.95	−1.84	n.d.	11	n.d.	42
3	**1**	−2.35	−4.03	15	33	n.d.	19
4	**1**	−2.35	−4.59	33	10	n.d.	17
5	**2**	−1.95	−3.61	74	3	n.d.	—
6	**2**	−1.95	−4.54	65	8	n.d.	—
7	**2**	−2.35	−7.17	88	2	n.d.	—
8	**2**	−2.35	−5.93	75	3	n.d.	—

[a] FE_{red} corresponds to the electron consumption to produce the active species $[L^{-\bullet}, Co^{II}]^-$ by reducing initial species $[L, Co^{II}]^0$ (see main text).

[b] n.d. indicates that the product was not detected.

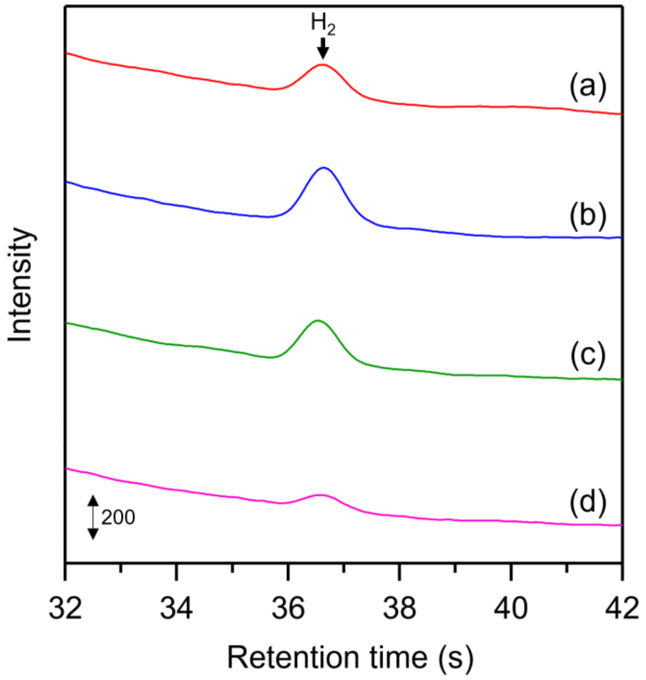

Figure S10. Chromatograms obtained by GC for (a) entry 2, (b) entry 4, (c) entry 6, and (d) entry 8 in the CPE experiments (see Table S3). Signal of H_2 (~36.5 s) is indicated by an arrow.

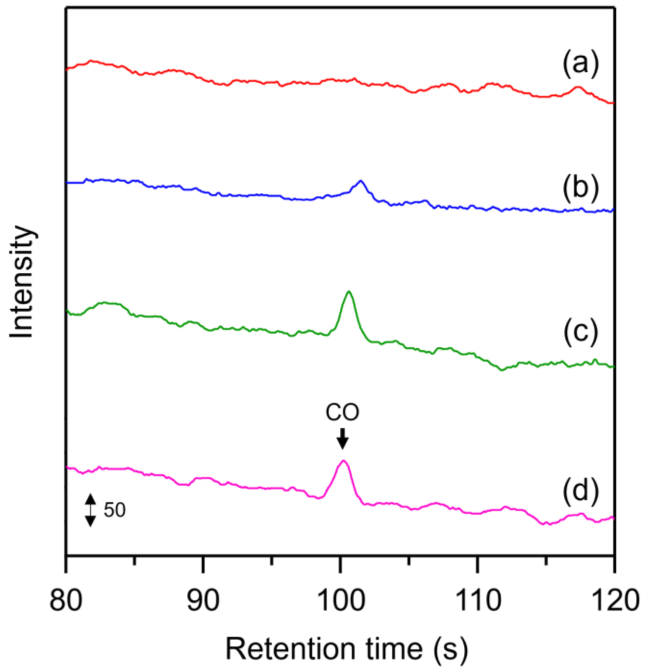

Figure S11. Chromatograms obtained by GC for (a) entry 2, (b) entry 4, (c) entry 6, and (d) entry 8 in the CPE experiments (see Table S3). Signal of CO (~100 s) is indicated by an arrow.

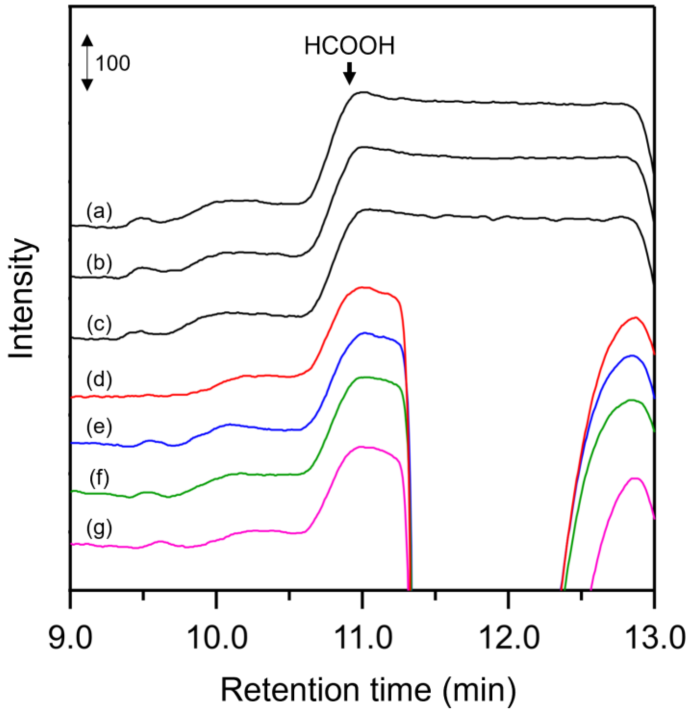

Figure S12. Chromatograms obtained by HPLC for (a) DMF containing HCOOH corresponding to the charge of 1.0 C in CPE experiments, (b) DMF containing HCOOH corresponding to the charge of 0.25 C in CPE experiments, (c) DMF, (d) catholyte of entry 2 of in CPE experiments, (e) catholyte of entry 4 in the CPE experiments, (f) catholyte of entry 6 in the CPE experiments, and (g) catholyte of entry 8 in the CPE experiments (see also Table S3). Signal of HCOOH (~11 min) is indicated by an arrow.

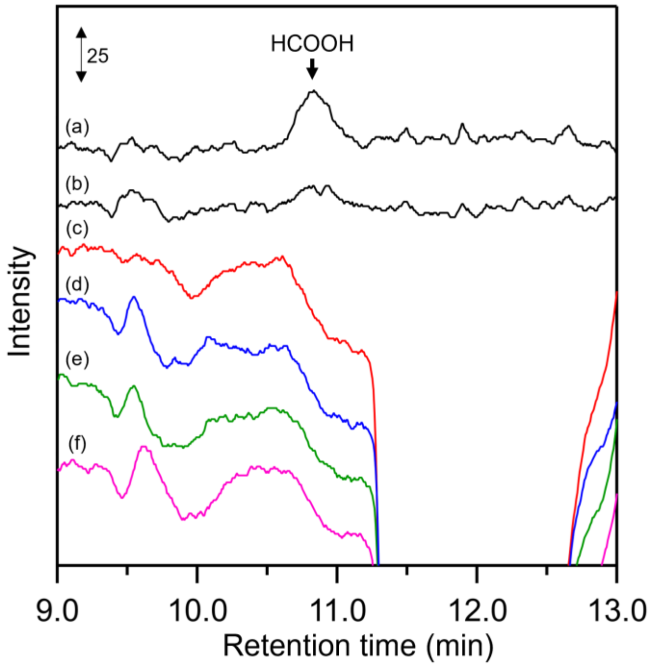

Figure S13. Chromatograms obtained by HPLC, where the chromatogram of pure DMF was subtracted, for (a) DMF containing HCOOH corresponding to the charge of 1.0 C in the CPE experiments, (b) DMF containing HCOOH corresponding to the charge of 0.25 C in the CPE experiments, (c) catholyte of entry 2 in the CPE experiments, (d) catholyte of entry 4 in the CPE experiments, (e) catholyte of entry 6 in the CPE experiments, and (f) catholyte of entry 8 in the CPE experiments (see also Table S3). Signal of HCOOH (~11 min) is indicated with arrows.

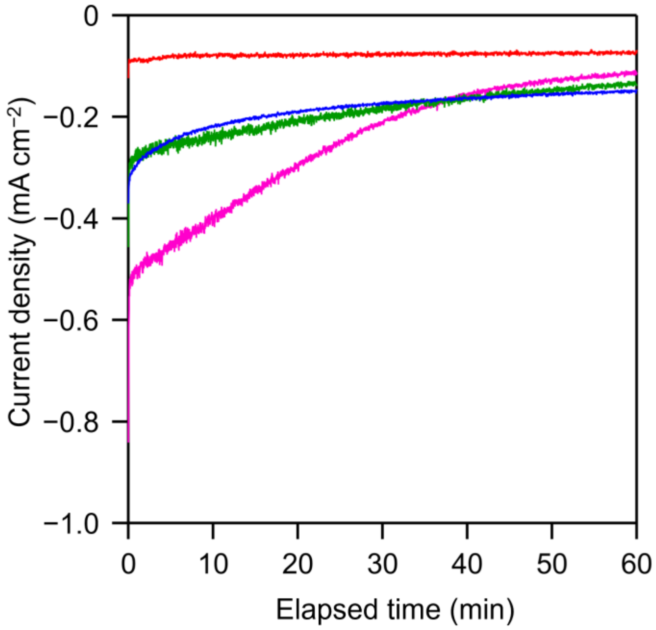

Figure S14. Time dependence of current density during CPE for entry 2 (red), entry 4 (blue), entry 6 (green), and entry 8 (pink).

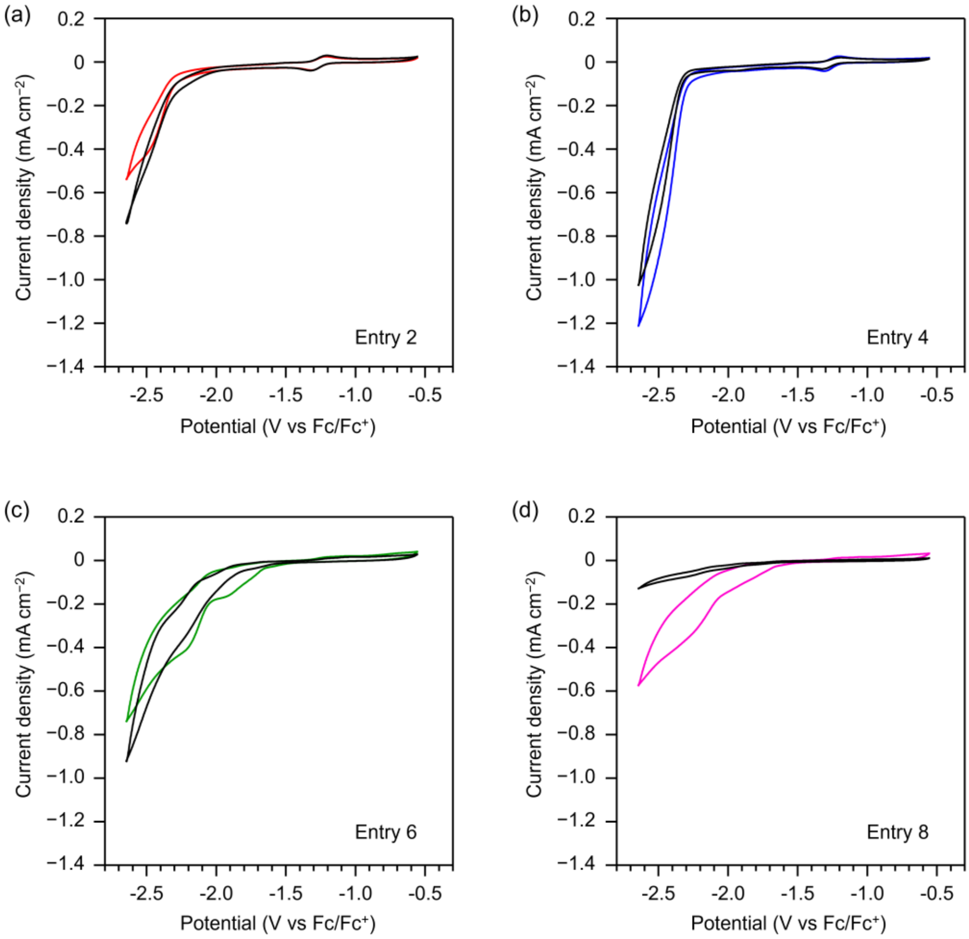

Figure S15. CV curves recorded before (colored) and after (black) the CPE for (a) entry 2, (b) entry 4, (c) entry 6, and (d) entry 8.

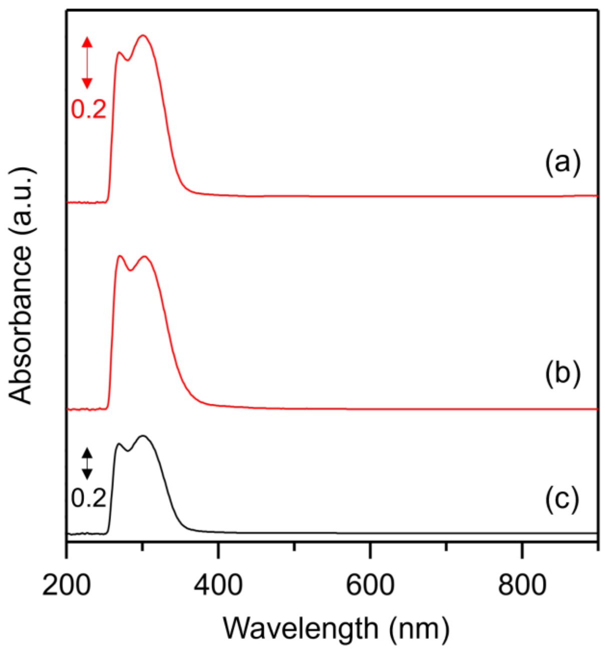

Figure S16. UV-vis spectra of catholyte (a) before (DMF:2,2,2-trifluoroethanol (9:1) solution containing 0.5 mM **2** and 0.1 M $TBAClO_4$) and (b) after CPE using **2** at −1.95 V. (c) UV-vis spectrum of DMF solution containing 0.5 mM **2** for reference.

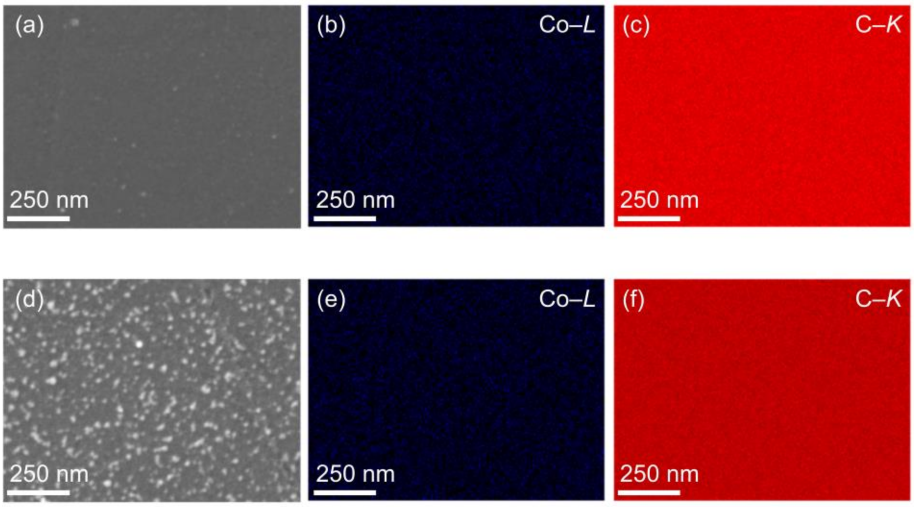

(g)

Entry	Atomic percent (%)			
	Co	O	N	C
Non-soaked area	0.00	0.73	0.00	99.27
Soaked area	0.01	0.83	0.00	99.16

Figure S17. SEM–EDX analysis of a glassy carbon electrode after CPE at −1.95 V vs. Fc/Fc^+ using **2**. (a)–(c) are data for the area non-soaked into the electrolytes, and (d)–(f) are for the area soaked into the electrolyte. (a) and (d) are SEM images. (b) and (e) are SEM-EDX maps for Co-*L*. (c) and (f) are SEM-EDX maps for C-*K*. (g) is the summary of the SEM-EDX analysis. It reveals no obvious formation of cobalt nanoparticles during the CPE.

S9. DFT calculations

S9.1 Consideration of catalytic cycles based on the ability of CO_2 binding

In the main text, we concluded that the catalytic cycles of **1** and **2** follows C→E→E and 2E→C manners, respectively, based on the electrochemical studies, where C and E represent CO_2 binding and electron transfer, respectively. The obtained catalytic cycles were also supported by the DFT calculations regarding the CO_2-binding ability of each reduction states (Figure S18), as described below.

As for **1**, precatalyst [L, Co^{II}]^0 exhibits no CO_2 binding (Figure S18 left), whereas, one-electron reduced [$L^{-\cdot}$, Co^{II}]$^-$ species exhibits the CO_2 binding with the binding energy of $\Delta G = 0.53$ kcal mol^{-1} (Figure S18 center). Here, the electron transfer from the ligand to Co ion is observed along with this CO_2 binding, i.e., formation of ^{1}[L, Co^I–CO_2]$^-$. The left superscript indicates the spin multiplicity. In this state, CO_2 molecules exhibits bending (∠OCO = 133°), indicating the activation. Notably, the CO_2-binding energy is reduced from 0.53 kcal mol^{-1} to −2.1 kcal mol^{-1} by the further reduction to [L, Co^0]$^{2-}$ (Figure S18 right), which is consistent with the experimental result exhibiting the significantly increased catalytic current in the potential range corresponding to [L, Co^0]$^{2-}$ (< −2.3 V in Figure 1c). As for **2**, as-prepared [L, Co^{II}, Co^{II}]$^{2+}$ exhibits no CO_2 binding (Figure S18 left). However, one-electron reduced [L, $Co^{1.5}$, $Co^{1.5}$]$^+$, which is not observed in CV because of the one-step two-electron reduction of [L, Co^{II}, Co^{II}]$^{2+}$, and the two-electron reduced state ^{5}[L, Co^I, Co^I]^0 can exhibits the CO_2 binding with the binding energy of 4.6 and 0.77 kcal mol^{-1}, respectively (Figure S18 center and right). In ^{5}[L, Co^I, Co^I–CO_2]^0, ∠OCO was 143°.

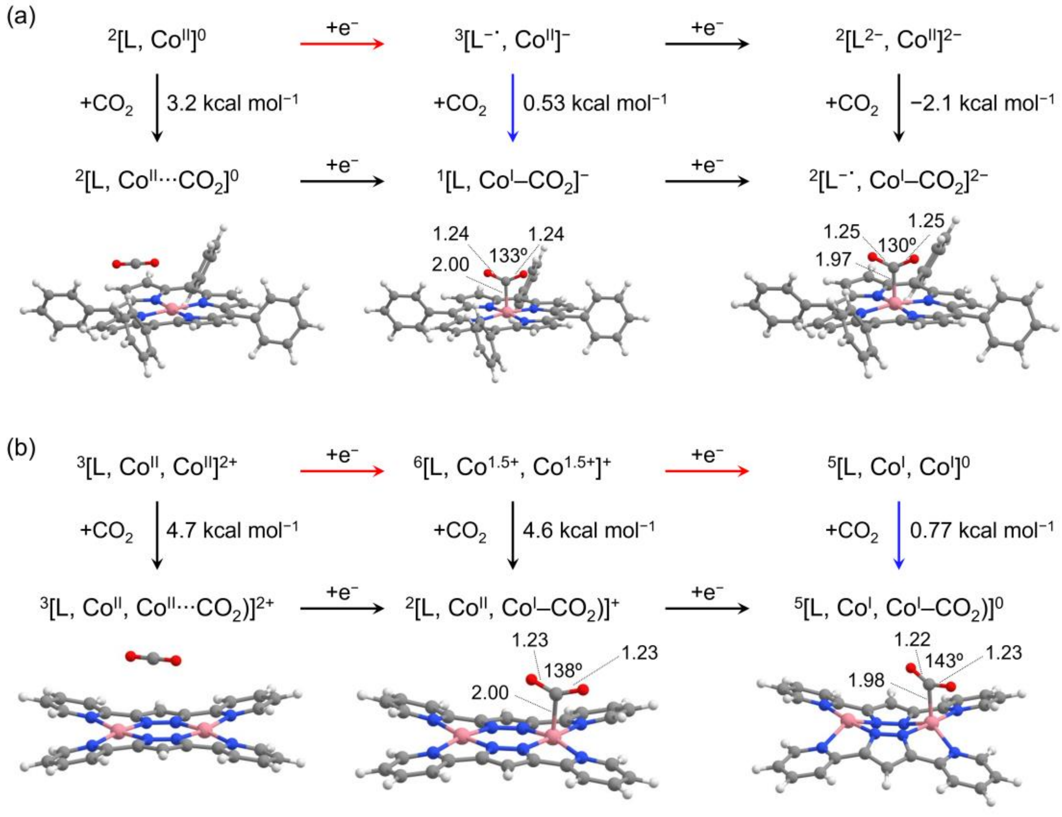

Figure S18. Square scheme related to the CO_2 binding for (a) **1** and (b) **2**, showing the difference in the Gibbs energy with and without CO_2 binding obtained by DFT calculations. DFT-optimized molecular structures coexisting with CO_2 are shown at the bottom. Selected bond lengths in Ångström unit and ∠OCO angles are shown at the bottom. Color code: C, gray; H, white; N, blue; Co, pink; O, red. Colored arrows represent the plausible pathway at around the onset potential.

S9.2 List of the computational results

Table S4. Electronic energy (*E*), Gibbs free energy (*G*), and relative *G* among the species with the same charges for the optimized structures of $Co_2(bpypz)_2$ complex with various charges and spin multiplicities. Mulliken charges and spin densities on the Co ions and ligand are also listed. The optimized structures are shown in the following Figures S19 and S20.

$Co_2(bpypz)_2$						
Charge, Multiplicity	*E* / Hartree	*G* / Hartree	Relative *G* / kcal mol^{-1}	Charge, Spin density		
				Co_L[a]	Co_R[a]	Ligand
2, 1	−4205.15001969	−4204.799785	17	0.236, −1.06	0.236, 1.06	1.528, 0.00
2, 3	−4205.17688639	−4204.82764	0	0.266, 1.07	0.266, 1.07	1.468, −0.13
2, 5	−4205.17231547	−4204.826717	0.58	0.439, 2.78	0.282, 1.07	1.279, 0.15
2, 7	−4205.16115887	−4204.819096	5.4	0.746, 2.79	0.746, 2.79	0.508, 0.42
1, 2	−4205.28812179	−4204.940065	11	0.001, −0.01	0.194, 1.06	0.805, −0.06
1, 4	−4205.29831384	−4204.956462	0.98	0.243, 1.09	0.199, 2.04	0.559, −0.13
1, 6	−4205.29543946	−4204.958026	0	0.598, 2.49	0.598, 2.49	−0.196, 0.02
0, 1	−4205.41238301	−4205.065563	5.5	−0.059, 0.00	−0.059, 0.00	0.117, 0.00
0, 3	−4205.37698205	−4205.033207	26	0.039, 0.61	0.040, 0.61	−0.079, 0.79
0, 5	−4205.41200339	−4205.074313	0	0.299, 1.96	0.299, 1.96	−0.599, 0.08
−1, 2	−4205.50162923	−4205.159028	0	−0.148, −0.10	−0.148, −0.10	−0.704, 1.20
−1, 4	−4205.49271357	−4205.156442	1.6	−0.175, −0.25	0.218, 2.07	−1.043, 1.18
−1, 6	−4205.47247849	−4205.141531	11	0.184, 2.05	0.102, 1.16	−1.286, 1.79
−2, 1	−4205.57655424	−4205.236953	0	−0.225, 0.21	−0.225, −0.21	−1.550, 0.00
−2, 3	−4205.57149206	−4205.234895	1.3	−0.174, −0.26	−0.174, −0.26	−1.652, 2.52
−2, 5	−4205.52806428	−4205.194052	27	−0.083, 0.58	−0.083, 0.58	−1.833, 2.85

[a] Co_L and Co_R correspond to Co sites depicted in left and right, respectively, in the following Figures S19 and S20.

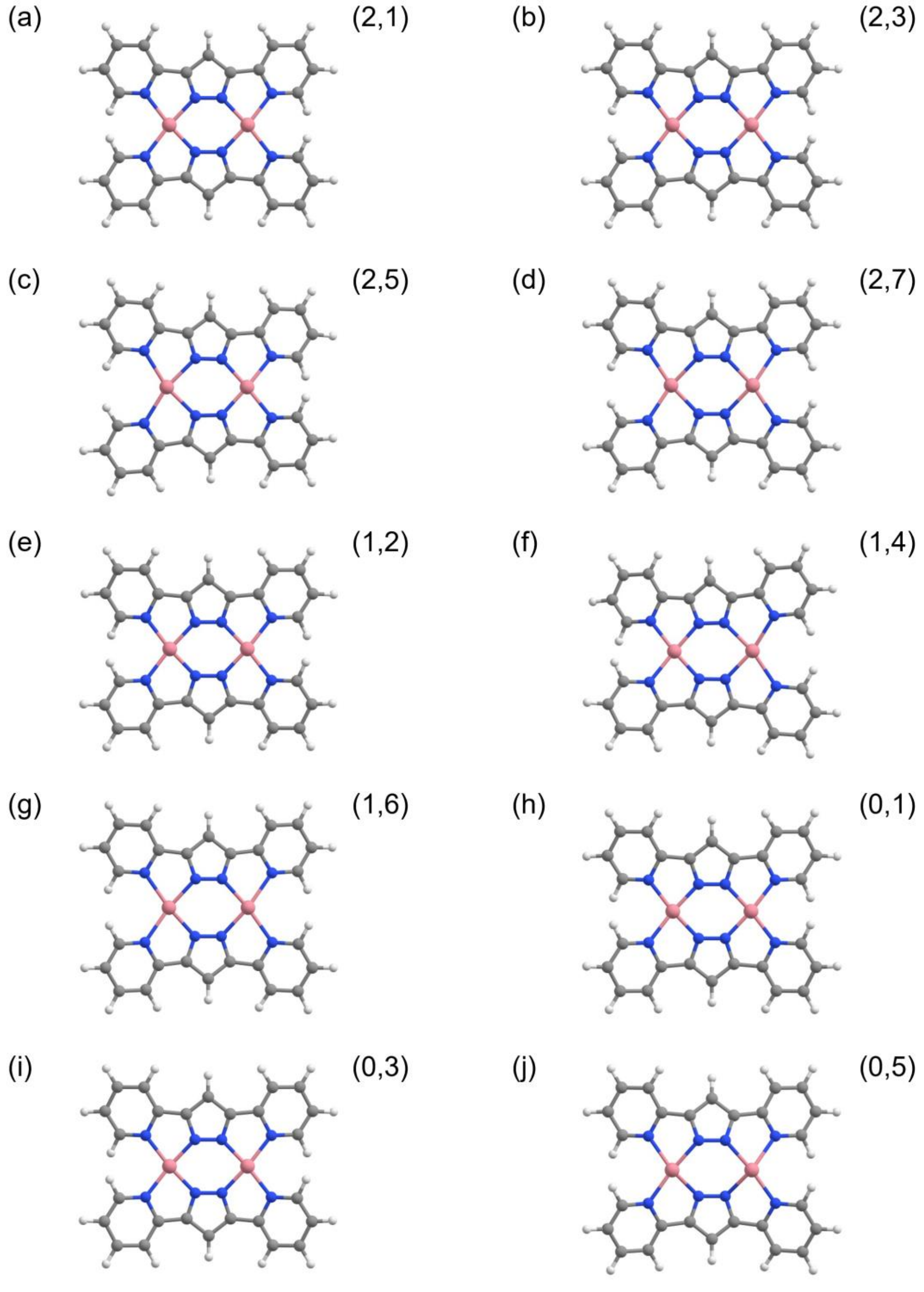

Figure S19. Optimized structures of $Co_2(bpypz)_2$ complex with (charge,spin multiplicity) = (a) (2,1), (b) (2,3), (c) (2,5), (d) (2,7), (e) (1,2), (f) (1,4), (g) (1,6), (h) (0,1), (i) (0,3), and (j) (0,5).

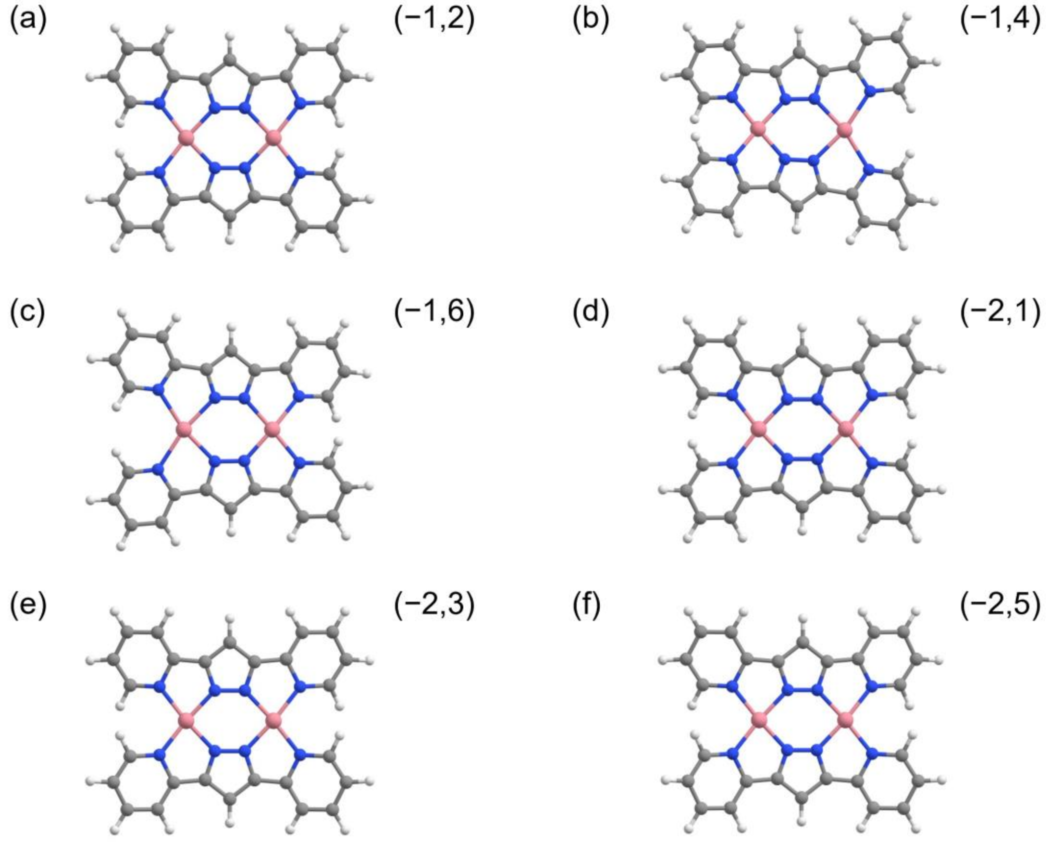

Figure S20. Optimized structures of $Co_2(bpypz)_2$ complex with (charge,spin multiplicity) = (a) (−1,2), (b) (−1,4), (c) (−1,6), (d) (−2,1), (e) (−2,3), and (f) (−2,5).

Table S5. Electronic energy (*E*), Gibbs free energy (*G*), and relative *G* among the species with the same charges for the optimized structures of Co_2(bpypz)$_2$ complex coexisting with a CO_2 molecule with various charges and spin multiplicities. Mulliken charges and spin densities on the Co ions, ligand, and CO_2 fragment are also listed. The optimized structures are shown in the following Figure S21.

Co_2(bpypz)$_2$ + CO_2							
Charge, Multiplicity	*E* / Hartree	*G* / Hartree	Relative *G* / kcal mol^{-1}	Charge, Spin density			
				Co_L[a]	Co_R[a]	Ligand	CO_2
2, 1	−4393.77471845	−4393.419152	0.86	−0.298, −1.06	−0.326, 1.06	2.505, 0.00	0.119, 0.00
2, 3	−4393.77505385	−4393.420522	0	−0.150, 1.06	−0.091, 1.05	2.140, −0.12	0.102, 0.00
2, 5	−4393.77028230	−4393.418414	1.3	0.421, 2.78	−0.412, 1.06	1.886, 0.16	0.105, 0.00
2, 7	−4393.76375023	−4393.415218	3.3	0.370, 2.78	0.208, 2.78	1.331, 0.43	0.090, 0.00
1, 2	−4393.90760947	−4393.550973	0	0.290, 1.07	−2.216, −0.01	3.106, −0.07	−0.180, 0.00
1, 4	−4393.90183521	−4393.548683	1.4	0.566, 2.78	−1.959, 0.00	2.641, 0.22	−0.249, 0.00
1, 6	−4393.89598959	−4393.548277	1.7	0.607, 2.76	−0.848, 2.18	1.458, 0.39	−0.217, −0.33
0, 1	−4394.02443507	−4393.668286	3.2	0.046, 0.00	−2.362, 0.00	2.516, 0.00	−0.200, 0.00
0, 3	−4394.02215180	−4393.672982	0.27	0.433, 2.03	−2.351, 0.01	2.184, −0.04	−0.266, 0.00
0, 5	−4394.01793242	−4393.673417	0	0.341, 1.95	−0.636, 2.11	0.708, 0.22	−0.413, −0.29

[a] Co_L and Co_R correspond to Co sites depicted in left and right, respectively, in the following Figure S21.

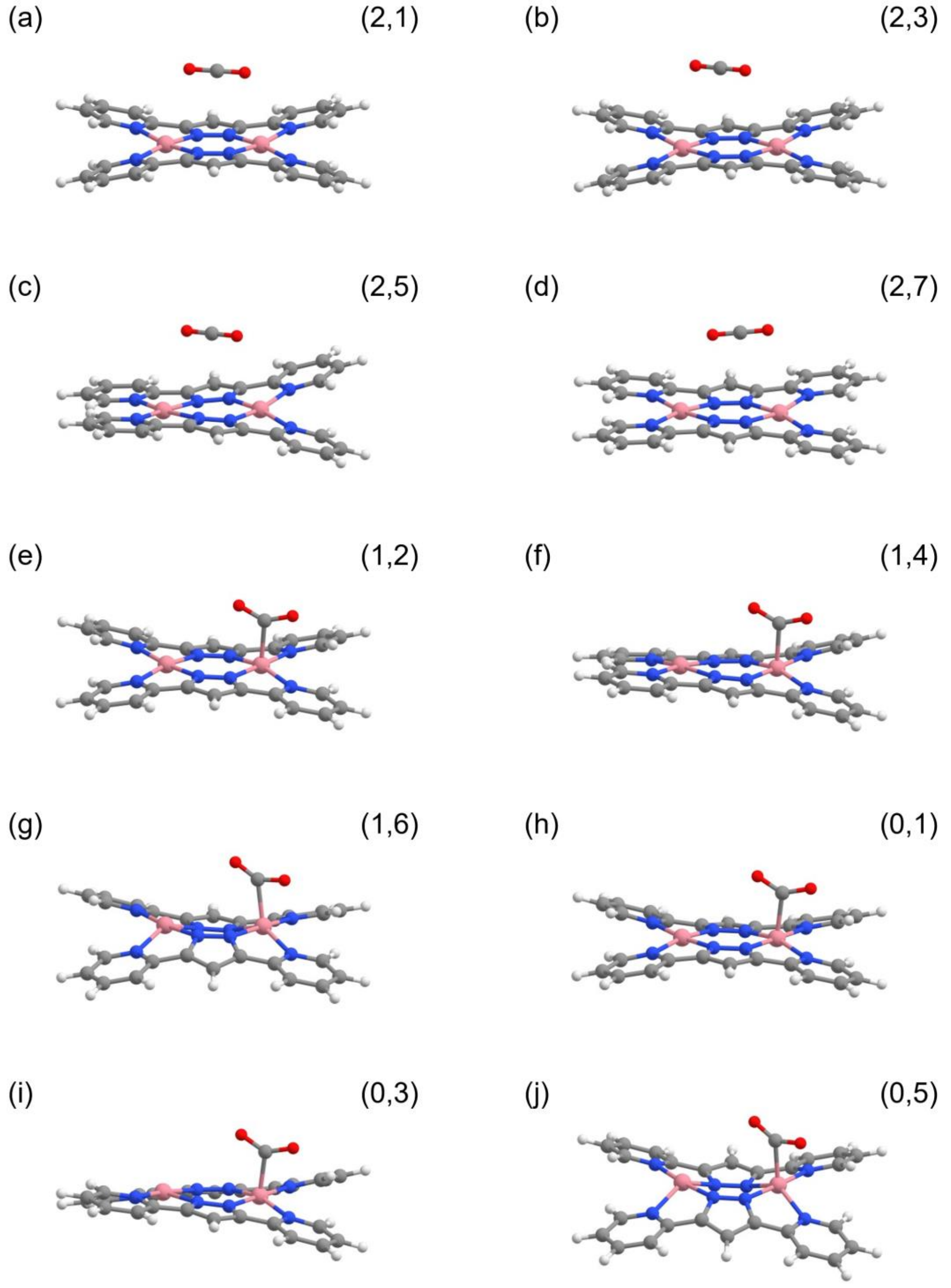

Figure S21. Optimized structures of $Co_2(bpypz)_2$ complexes coexisting with a CO_2 molecule with (charge,spin multiplicity) = (a) (2,1), (b) (2,3), (c) (2,5), (d) (2,7), (e) (1,2), (f) (1,4), (g) (1,6), (h) (0,1), (i) (0,3), and (j) (0,5).

Table S6. Electronic energy (*E*), Gibbs free energy (*G*), and relative *G* among the species with the same charges for the optimized structures of $Co_2(bpypz)_2$ complex coexisting with a CO_2H fragment with various charges and spin multiplicities. Mulliken charges and spin densities on the Co ions, ligand, and CO_2H fragment are also listed. The optimized structures are shown in the following Figure S22.

$Co_2(bpypz)_2 + CO_2H$							
Charge, Multiplicity	*E* / Hartree	*G* / Hartree	Relative *G* / kcal mol^{-1}	Charge, Spin density			
				Co_L[a]	Co_R[a]	Ligand	CO_2H
3, 1	−4394.07955063	−4393.709656	15	0.316, 1.02	−2.324, −0.05	4.388, −0.99	0.619, 0.03
3, 3	−4394.10323080	−4393.734282	0	0.222, 1.88	−2.540, −0.01	4.673, 0.13	0.646, 0.00
3, 5	−4394.09380328	−4393.72704	4.5	0.650, 2.80	−2.064, 0.02	3.908, 1.19	0.507, 0.00
3, 7	−4394.06656611	−4393.705092	18	0.800, 2.77	−1.895, 1.83	3.797, 1.39	0.299, 0.02
2, 2	−4394.32814248	−4393.957695	8.5	0.259, 1.07	−2.473, 0.00	3.635, −0.07	0.579, 0.00
2, 4	−4394.33770753	−4393.971271	0	0.510, 2.79	−2.092, 0.00	3.122, 0.21	0.460, 0.00
2, 6	−4394.31056024	−4393.948887	14	0.800, 2.76	−2.004, 1.76	2.975, 0.38	0.230, 0.09
1, 1	−4394.48363374	−4394.118791	7.9	0.366, 1.07	−2.236, −0.99	2.806, −0.12	0.064, 0.05
1, 3	−4394.48010425	−4394.11534	10	0.309, 1.06	−2.229, 0.99	2.889, −0.01	0.031, −0.04
1, 5	−4394.49522459	−4394.13145	0	0.788, 2.71	−1.465, 0.96	1.816, 0.36	−0.139, −0.03

[a] Co_L and Co_R correspond to Co sites depicted in left and right, respectively, in the following Figure S22.

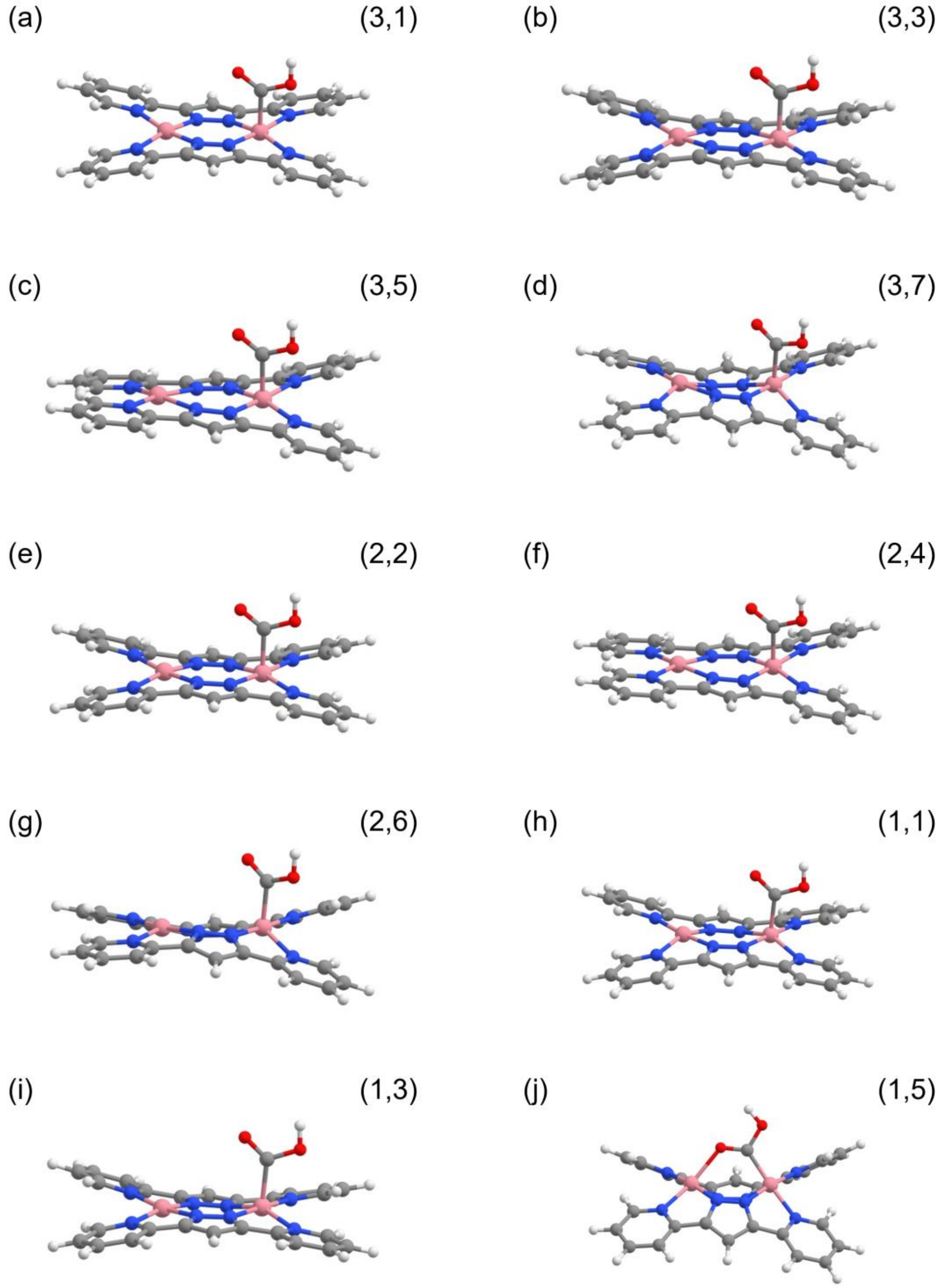

Figure S22. Optimized structures of $Co_2(bpypz)_2$ complexes coexisting with a CO_2H fragment with (charge,spin multiplicity) = (a) (3,1), (b) (3,3), (c) (3,5), (d) (3,7), (e) (2,2), (f) (2,4), (g) (2,6), (h) (1,1), (i) (1,3), and (j) (1,5).

Table S7. Electronic energy (E), Gibbs free energy (G), and relative G among the species with the same charges for the optimized structures of $Co_2(bpypz)_2$ complex coexisting with a CO molecule with various charges and spin multiplicities. Mulliken charges and spin densities on the Co ions, ligand, and CO fragment are also listed. The optimized structures are shown in the following Figures S23 and S24.

$Co_2(bpypz)_2$ + CO							
Charge, Multiplicity	E / Hartree	G / Hartree	Relative G / kcal mol^{-1}	Charge, Spin density			
				Co_L[a]	Co_R[a]	Ligand	CO
4, 1	−4318.00662018	−4317.653655	6.9	0.387, 1.86	−1.256, −1.63	4.753, −0.18	0.116, −0.05
4, 3	−4318.01283590	−4317.660731	2.4	0.465, 1.22	−1.152, −1.01	4.601, 1.82	0.086, −0.02
4, 5	−4318.01174912	−4317.663132	0.92	0.889, 2.80	−1.325, −0.95	4.290, 2.17	0.146, −0.02
4, 7	−4318.01291235	−4317.664602	0	0.891, 2.80	−1.354, 1.16	4.349, 2.00	0.113, 0.04
4, 9	−4318.00303056	−4317.657451	4.5	0.826, 2.79	−0.071, 2.69	3.297, 2.48	−0.052, 0.04
3, 2	−4318.27015720	−4317.918335	0	0.340, 1.86	−1.221, −1.02	3.810, 0.19	0.071, −0.03
3, 4	−4318.26654244	−4317.913174	3.2	0.645, 2.79	−1.613, 0.01	3.592, 0.20	0.376, 0.00
3, 6	−4318.26495525	−4317.916803	0.96	0.782, 2.79	−1.359, 1.55	3.448, 0.61	0.128, 0.05
3, 8	−4318.25230719	−4317.907076	7.1	0.778, 2.78	−0.190, 2.68	2.457, 1.51	−0.045, 0.04
2, 1	−4318.49291048	−4318.139359	9.0	0.369, 1.07	−1.213, −1.02	2.796, −0.03	0.047, −0.02
2, 3	−4318.49290745	−4318.140397	8.3	0.371, 1.06	−1.210, 1.02	2.793, −0.10	0.047, 0.02
2, 5	−4318.50166014	−4318.153696	0	0.667, 2.78	−1.255, 1.02	2.503, 0.18	0.086, 0.02
2, 7	−4318.49047044	−4318.147621	3.8	0.685, 2.78	−0.421, 2.69	1.699, 0.49	0.038, 0.03

[a] Co_L and Co_R correspond to Co sites depicted in left and right, respectively, in the following Figures S23 and S24.

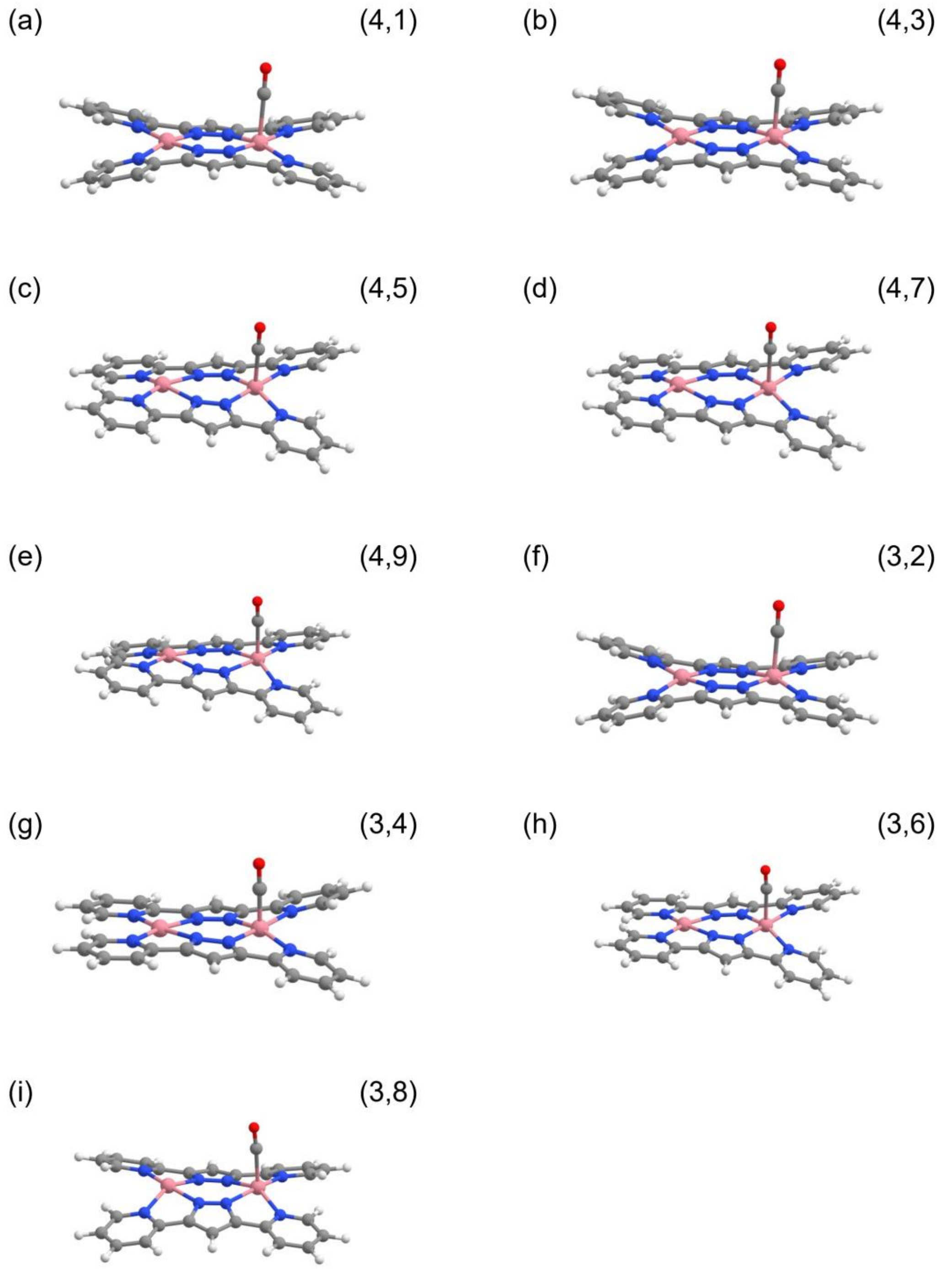

Figure S23. Optimized structures of $Co_2(bpypz)_2$ complexes coexisting with a CO molecule with (charge,spin multiplicity) = (a) (4,1), (b) (4,3), (c) (4,5), (d) (4,7), (e) (4,9), (f) (3,2), (g) (3,4), (h) (3,6), and (i) (3,8).

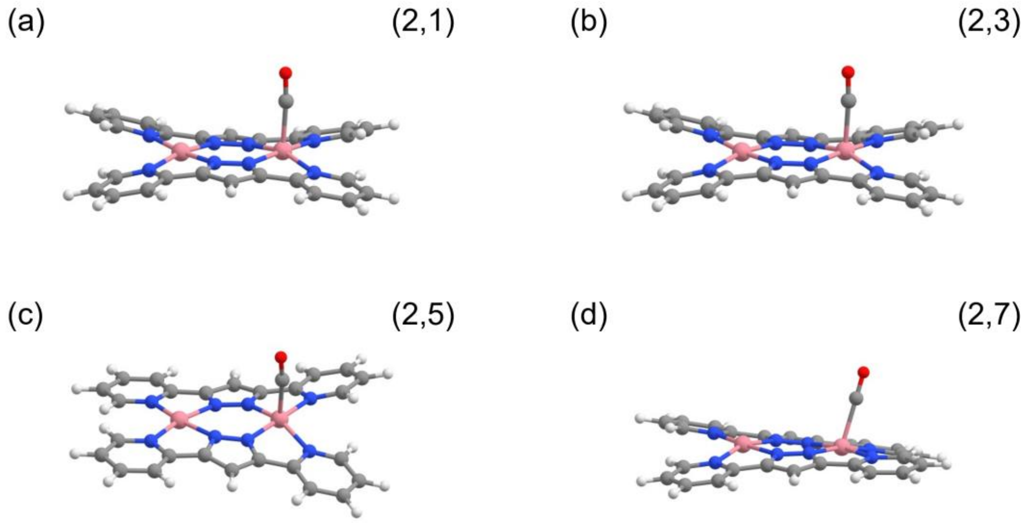

Figure S24. Optimized structures of $Co_2(bpypz)_2$ complexes coexisting with a CO molecule with (charge,spin multiplicity) = (a) (2,1), (b) (2,3), (c) (2,5), and (d) (2,7).

Table S8. Electronic energy (*E*), Gibbs free energy (*G*), and relative *G* among the species with the same charges for the optimized structures of Co tetraphenylporphyrin complex with various charges and spin multiplicities. Mulliken charges and spin densities on the Co ion and ligand are also listed. The optimized structures are shown in the following Figure S25.

Co tetraphenylporphyrin					
Charge, Multiplicity	*E* / Hartree	*G* / Hartree	Relative *G* / kcal mol^{-1}	Charge, Spin density	
				Co	Ligand
0, 2	−3295.60849223	−3295.084013	0	0.622, 1.04	−0.622, −0.04
0, 4	−3295.59518985	−3295.071489	7.9	0.901, 2.75	−0.901, 0.25
−1, 1	−3295.70955303	−3295.185854	4.0	0.221, 0.00	−1.221, −0.00
−1, 3	−3295.71175279	−3295.192256	0	0.561, 1.06	−1.561, 0.94
−2, 2	−3295.79989105	−3295.281024	0	0.364, 0.89	−2.364, 0.11
−2, 4	−3295.79412961	−3295.279301	1.1	0.492, 1.08	−2.492, 1.92
−3, 1	−3295.86283524	−3295.346297	1.0	0.301, 0.88	−3.301, −0.88
−3, 3	−3295.86388472	−3295.347952	0	0.289, 0.90	−3.289, 1.10

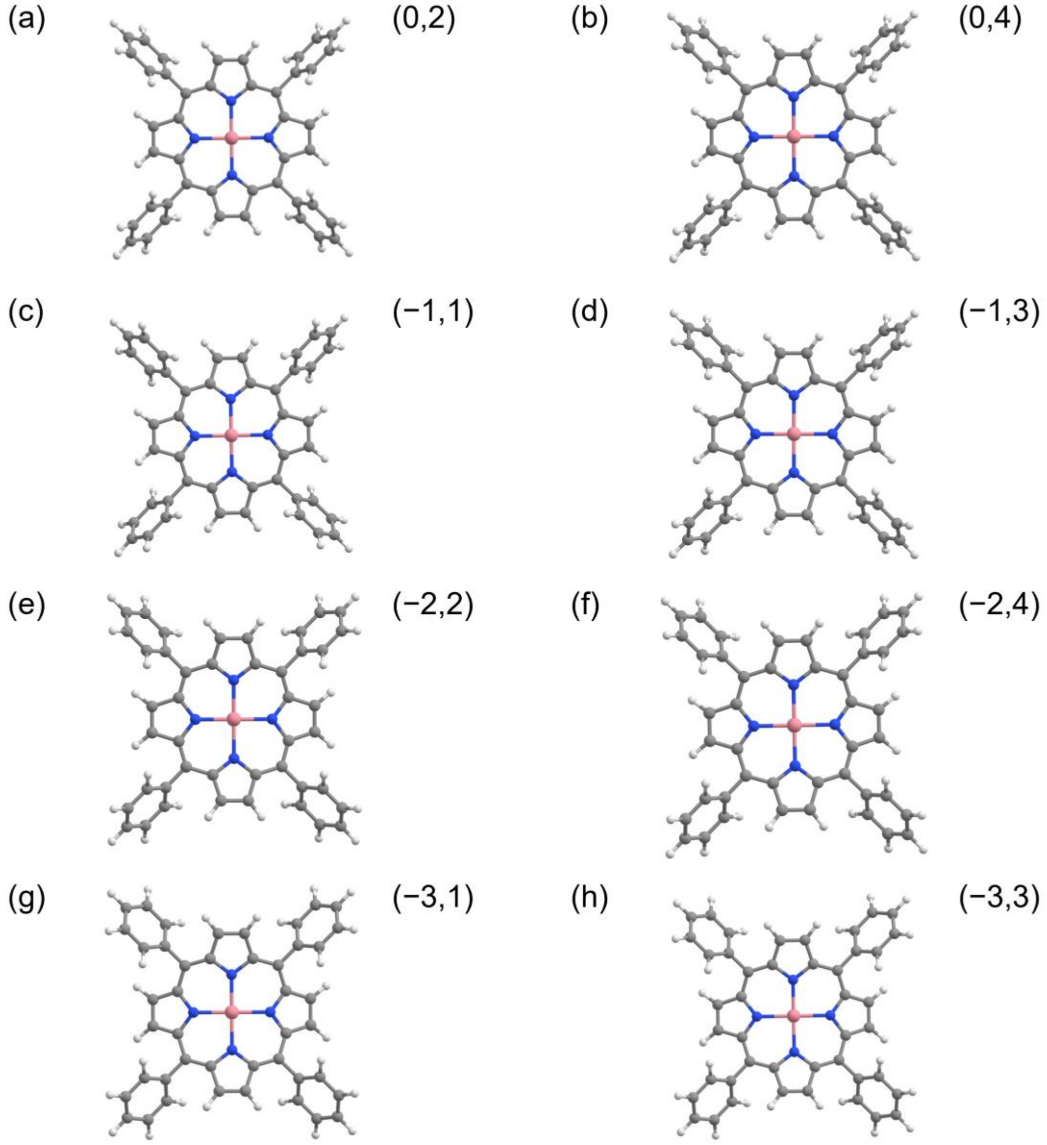

Figure S25. Optimized structures of Co tetraphenylporphyrin complexes with (charge,spin multiplicity) = (a) (0,2), (b) (0,4), (c) (−1,1), (d) (−1,3), (e) (−2,2), (f) (−2,4), (g) (−3,1), and (h) (−3,3).

Table S9. Electronic energy (E), Gibbs free energy (G), and relative G among the species with the same charges for the optimized structures of Co tetraphenylporphyrin complex coexisting with a CO_2 molecule with various charges and spin multiplicities. Mulliken charges and spin densities on the Co ion, ligand, and CO_2 fragment are also listed. The optimized structures are shown in the following Figure S26.

Co tetraphenylporphyrin + CO_2						
Charge, Multiplicity	E / Hartree	G / Hartree	Relative G / kcal mol^{-1}	Charge, Spin density		
				Co	Ligand	CO_2
0, 2	−3484.20751614	−3483.679185	0	0.352, 1.03	−0.525, −0.04	0.173, 0.00
0, 4	−3484.19415235	−3483.666175	8.2	0.826, 2.75	−0.985, 0.25	0.160, 0.00
−1, 1	−3484.31907831	−3483.787025	0	−0.799, 0.00	−0.095, 0.00	−0.106, 0.00
−1, 3	−3484.31083232	−3483.78466	1.5	0.180, 1.04	−1.350, 0.96	0.170, 0.00
−2, 2	−3484.41357688	−3483.88476	0	−0.962, 0.01	−0.885, 0.99	−0.153, −0.01
−2, 4	−3484.39343053	−3483.869346	9.7	0.166, 1.05	−2.329, 1.95	0.163, 0.01
−3, 1	−3484.49086180	−3483.963517	0	−1.076, 0.02	−1.723, −0.01	−0.201, −0.01
−3, 3	−3484.48861126	−3483.961344	1.4	−1.035, 0.00	−1.766, 2.00	−0.198, 0.00

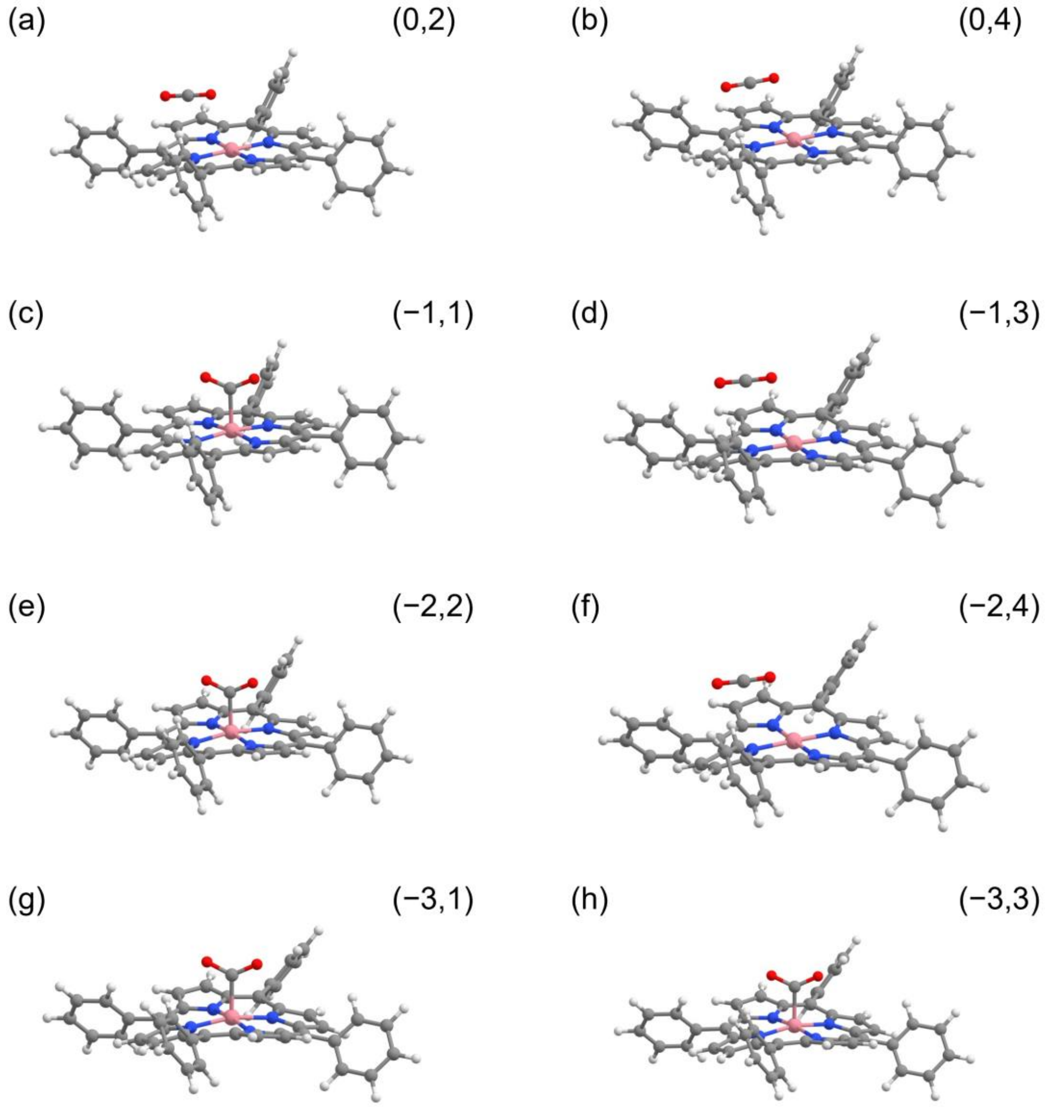

Figure S26. Optimized structures of Co tetraphenylporphyrin complexes coexisting with a CO_2 molecule with (charge,spin multiplicity) = (a) (0,2), (b) (0,4), (c) (−1,1), (d) (−1,3), (e) (−2,2), (f) (−2,4), (g) (−3,1), and (h) (−3,3).

Table S10. Electronic energy (*E*), Gibbs free energy (*G*), and relative *G* among the species with the same charges for the optimized structures of Co tetraphenylporphyrin complex coexisting with a CO_2H fragment with various charges and spin multiplicities. Mulliken charges and spin densities on the Co ion, ligand, and CO_2H fragment are also listed. The optimized structures are shown in the following Figure S27.

Co tetraphenylporphyrin + CO_2H						
Charge, Multiplicity	*E* / Hartree	*G* / Hartree	Relative *G* / kcal mol^{-1}	Charge, Spin density		
				Co	Ligand	CO_2H
1, 2	−3484.58279461	−3484.035577	0	−1.530, 0.06	1.805, 0.95	0.725, −0.02
1, 4	−3484.51405936	−3483.971028	41	−1.602, 0.13	1.885, 2.90	0.716, −0.02
0, 1	−3484.77912312	−3484.233809	0	−1.073, 0.00	0.493, 0.00	0.579, 0.00
0, 3	−3484.71511262	−3484.17258	38	−1.548, 0.07	0.880, 1.94	0.669, −0.01
−1, 2	−3484.88668934	−3484.345141	0	−1.222, −0.03	−0.301, 1.03	0.523, 0.01
−1, 4	−3484.82639552	−3484.287477	36	−1.248, 0.00	−0.292, 2.97	0.540, 0.03
−2, 1	−3484.97522983	−3484.44017	0	−1.282, 0.01	−1.198, 0.00	0.480, 0.00
−2, 3	−3484.97279356	−3484.434377	3.6	−1.279, −0.07	−1.211, 2.05	0.490, 0.02

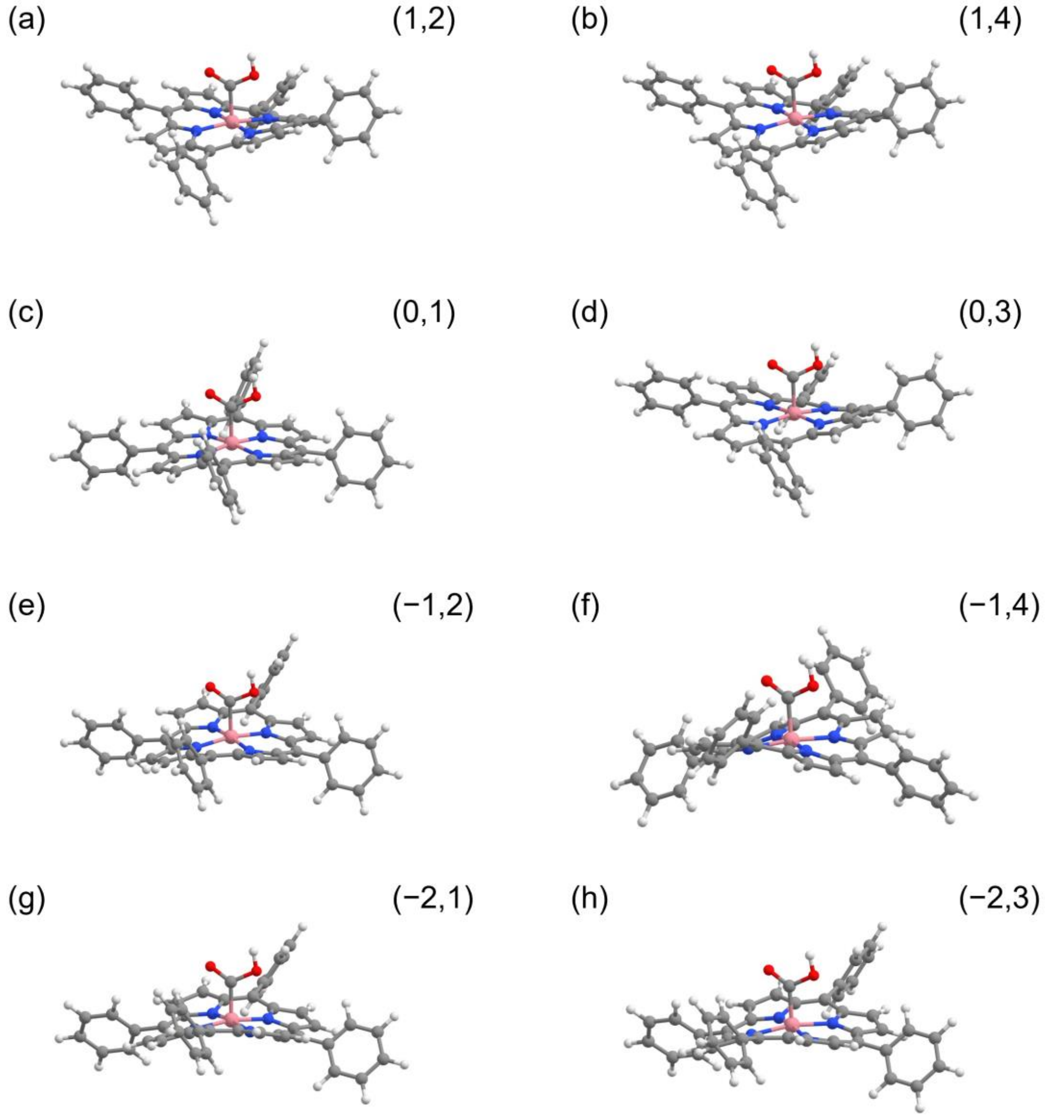

Figure S27. Optimized structures of Co tetraphenylporphyrin complexes coexisting with a CO_2H fragment with (charge,spin multiplicity) = (a) (1,2), (b) (1,4), (c) (0,1), (d) (0,3), (e) (−1,2), (f) (−1,4), (g) (−2,1), and (h) (−2,3).

Table S11. Electronic energy (E), Gibbs free energy (G), and relative G among the species with the same charges for the optimized structures of Co tetraphenylporphyrin complex coexisting with a CO molecule with various charges and spin multiplicities. Mulliken charges and spin densities on the Co ion, ligand, and CO fragment are also listed. The optimized structures are shown in the following Figure S28.

Co tetraphenylporphyrin + CO						
Charge, Multiplicity	E / Hartree	G / Hartree	Relative G / kcal mol^{-1}	Charge, Spin density		
				Co	Ligand	CO
2, 2	−3408.53542530	−3408.002869	0	−0.585, 0.97	2.376, 0.00	0.209, 0.02
2, 4	−3408.52219266	−3407.99442	5.3	−0.790, 1.05	2.565, 1.92	0.225, 0.04
2, 6	−3408.50902088	−3407.98586	11	−0.187, 2.74	1.999, 2.22	0.189, 0.04
1, 1	−3408.74933073	−3408.219504	0	−0.867, 0.91	1.653, −0.93	0.214, 0.02
1, 3	−3408.74434433	−3408.217423	1.3	−0.477, 1.00	1.282, 0.96	0.196, 0.04
1, 5	−3408.67517102	−3408.152397	42	−0.453, 1.05	1.283, 2.91	0.170, 0.04
0, 2	−3408.93487008	−3408.410831	0	−1.001, 0.99	0.758, −0.01	0.243, 0.03
0, 4	−3408.91037938	−3408.386247	15	−0.148, 2.60	0.040, 0.37	0.107, 0.03
−1, 1	−3409.03862857	−3408.514926	1.1	−1.028, 1.00	−0.379, −1.02	0.407, 0.02
−1, 3	−3409.03976984	−3408.516712	0	−1.155, 1.00	−0.222, 0.97	0.377, 0.02

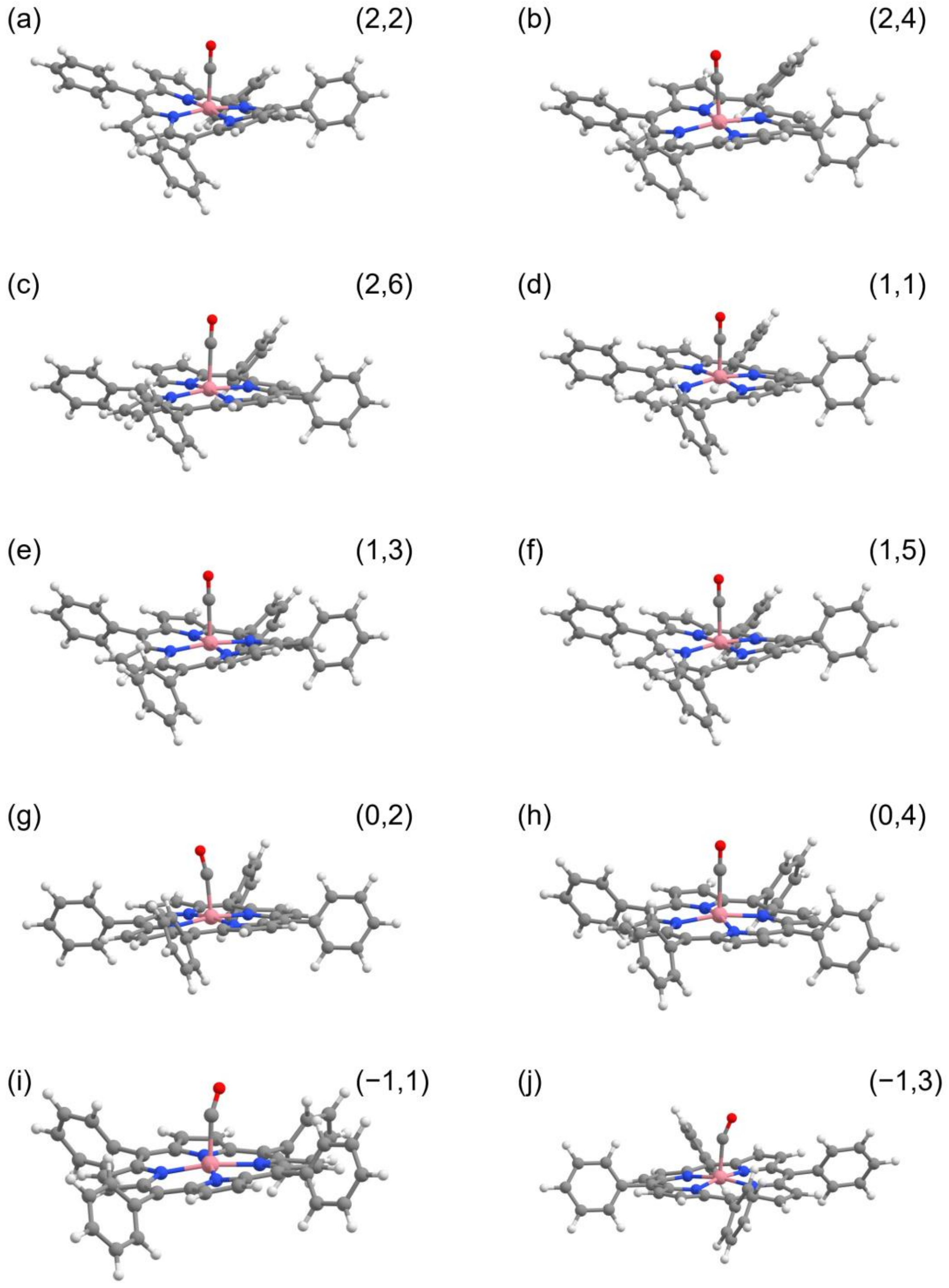

Figure S28. Optimized structures of Co tetraphenylporphyrin complexes coexisting with a CO molecule with (charge,spin multiplicity) = (a) (2,2), (b) (2,4), (c) (2,6), (d) (1,1), (e) (1,3), (f) (1,5), (g) (0,2), (h) (0,4), (i) (−1,1), and (j) (−1,3)

S10. References

[S1] Casabo, J., Pons, J., Siddiqi, K. S., *J. Chem. Soc. Dalton Trans*. **1989**, 1401.

[S2] Chadghan, A., Pons, J., Caubet, A., Casabó, J., Ros, J., Alvarez-Larena, A., Francesc Piniella, J., *Polyhedron* **2000,** *19*, 855–862.

[S3] Sheldrick, G. M., *Acta Cryst.* **2015**, *A71*, 3–8.

[S4] Sheldrick, G. M., *Acta Cryst.* **2015**, *C71*, 3–8.

[S5] Dolomanov, O. V., Bourhis, L. J., Gildea, R. J., Howard, J. A. K., Puschmann, H., *J. Appl. Cryst.* **2009**, *42*, 339–341.

[S6] Abdul, M., Nishiumi, T., Aoki, K., *J. Electroanal. Chem.* **2007**, *601*, 139.

[S7] (a) Becke, A. D. *Phys. Rev. A*, **1988**, *38*, 3098–3100 (b) Lee, C., Yang, W., Parr, R. G. *Phys. Rev. B*, **1988**, *37*, 785–789. (c) Becke, A. D., *J. Chem. Phys.* **1993**, *98*, 5648–5652.

[S8] (a) Schwabe, T., Grimme, S., *Phys. Chem. Chem. Phys.* **2007**, *9*, 3397–3406. (b) Grimme, S., Antony, J., Ehrlich, S., Krieg, H., *J. Chem. Phys.* **2010**, *132*, 154104.

[S9] Tomasi, J., Mennucci, B., Cammi, R., *Chem. Rev.* **2005**, *105*, 2999–3093.

[S10] Gaussian 16, Revision C.01, Frisch, M. J., Trucks, G. W., Schlegel, H. B., Scuseria, G. E., Robb, M. A., Cheeseman, J. R., Scalmani, G., Barone, V., Petersson, G. A., Nakatsuji, H., Li, X., Caricato, M., Marenich, A. V., Bloino, J., Janesko, B. G., Gomperts, R., Mennucci, B., Hratchian, H. P., Ortiz, J. V., Izmaylov, A. F., Sonnenberg, J. L., Williams-Young, D., Ding, F., Lipparini, F., Egidi, F., Goings, J., Peng, B., Petrone, A., Henderson, T., Ranasinghe, D., Zakrzewski, V. G., Gao, J., Rega, N., Zheng, G., Liang, W., Hada, M., Ehara, M., Toyota, K., Fukuda, R., Hasegawa, J., Ishida, M., Nakajima, T., Honda, Y., Kitao, O., Nakai, H., Vreven, T., Throssell, K., Montgomery, J. A., Jr., Peralta, J. E., Ogliaro, F., Bearpark, M. J., Heyd, J. J., Brothers, E. N., Kudin, K. N., Staroverov, V. N., Keith, T. A., Kobayashi, R., Normand, J., Raghavachari, K., Rendell, A. P., Burant, J. C., Iyengar, S. S., Tomasi, J., Cossi, M., Millam, J. M., Klene, M., Adamo, C., Cammi, R., Ochterski, J. W., Martin, R. L., Morokuma, K., Farkas, O., Foresman, J. B., Fox, D. J., Gaussian, Inc., Wallingford CT, 2016.

[S11] (a) Wachters, A. J. H., *J. Chem. Phys.* **1970**, *52*, 1033–1036. (b) Hay, P. J., *J. Chem. Phys.* **1977**, *66*, 4377–4384. (c) Raghavachari, K., Trucks, G. W., *J. Chem. Phys.* **1989**, *91*, 1062–1065.

[S12] (a) Ditchfield, R., Hehre, W. J., Pople, J. A., *J. Chem. Phys.* **1971**, *54*, 724–728. (b) Hehre, W. J., Ditchfield, R., Pople, J. A., *J. Chem. Phys.* **1972**, *56*, 2257–2261. (c) Hariharan, P. C., Pople, J. A., *Theoret. Chim. Acta* **1973**, *28*, 213–222. (d) Clark, T., Chandrasekhar, J., Spitznagel, G. W., Schleyer, P. V. R., *J. Comput. Chem.* **1983**, *4*, 294–301.

[S13] Kahn, O. *Molecular Magnetism*, Wiley-VCH, New York, 1993.

[S14] Costentin, C., Drouet, S., Robert, M., Savéant, J.-M., *J. Am. Chem. Soc.*, **2012**, *134*, 11235–11242.

[S15] Nichols, E. M.; Derrick, J. S.; Nistanaki, S. K.; Smith, P. T.; Chang, C. J., *Chem. Sci.* **2018,** *9*, 2952–2960.

[S16] Nie, W.; Wang, Y.; Zheng, T.; Ibrahim, A.; Xu, Z.; McCrory, C. C. L., *ACS Catal.* **2020,** *10*, 4942–4959.

[S17] Tsubonouchi, Y.; Takahashi, D.; Berber, M. R.; Mohamed, E. A.; Zahran, Z. N.; Alenad, A. M.; Althubiti, N. A.; Yagi, M., *Electrochim. Acta* **2021,** *387*, 138545.

[S18] Elgrishi, N.; Chambers, M. B.; Artero, V.; Fontecave, M., *Phys. Chem. Chem. Phys.* **2014,** *16*, 13635–13644.

[S19] Bairagi, A.; Pereverzev, A. Y.; Tinnemans, P.; Pidko, E. A.; Roithova, J., *J. Am. Chem. Soc.* **2024,** *146*, 5480–5492.
